# Prevalence and distribution of low and high myopia in Mexican outpatients: a nationwide cross-sectional clinic-based study

**DOI:** 10.3389/fmed.2026.1753396

**Published:** 2026-02-23

**Authors:** José Antonio Magaña-Lizárraga, Abraham García-Gil, José Manuel Romero-Flores, Eduardo Espinoza-Angulo, Héctor Machado-Jiménez, Marco Antonio Luna-Ruiz-Esparza, Humberto Gómez-Campaña, Leticia Riverón-Negrete, Linda Nasser-Nasser, Abraham Campos-Romero, Jonathan Alcántar-Fernández

**Affiliations:** 1 Innovation and Research Department, Salud Digna, Culiacan, Mexico; 2 Optometry Department, Salud Digna, Culiacan, Mexico; 3 Medical Direction, Salud Digna, Culiacan, Mexico; 4 Unidad de Genética de la Nutrición, Instituto de Investigaciones Biomédicas, Universidad Nacional Autónoma de México/Instituto Nacional de Pediatría, Mexico City, Mexico; 5 Medical Direction, Visión Cirugía Ambulatoria S.C., Monterrey, Mexico

**Keywords:** adults, astigmatism, children and adolescents, epidemiology, high myopia, myopia, myopia severity, prevalence

## Abstract

**Background:**

Myopia is a global public health concern and a leading cause of distance vision impairment, affecting mainly children and young adults. Although several studies have reported myopia prevalence in Mexico, recent nationwide data, especially regarding high myopia, are lacking following the COVID-19 pandemic. We estimated the prevalence and distribution of low and high myopia in a cohort of Mexican outpatients.

**Methods:**

A retrospective, cross-sectional, clinic-based study was conducted using anonymized electronic health records from Salud Digna clinics. Data included individuals aged 6–100 years undergoing routine non-cycloplegic eye examinations between January and December 2023. Myopia was defined as spherical equivalent refraction (SER) ≤ −0.50 diopters (D) in the right eye and subclassified into low (−6.0 D < SER ≤ −0.50 D) or high (SER ≤ −6.0 D). Age-standardized prevalence rates were estimated overall and by sex and geographic location. Multinomial logistic regression evaluated potential risk between demographic/clinical background and myopia severity.

**Results:**

From 3,507,826 records, 1,337,526 (38.13%) individuals were myopic (median age: 28 years; 61.4% females). Age-standardized rates for total, low, and high myopia were 44.44% (95% CI, 44.36–44.52%), 43.31% (95% CI, 43.24–43.39%), and 1.12% (95% CI, 1.11–1.14%), respectively. Low myopia was more prevalent in males, whereas high myopia predominated in females (both *p* < 0.001). Both forms were most prevalent in individuals ≤ 10 through 31–40 years, peaking at 64.65 and 1.75% in the 21–30 age group. The Central region, specifically Mexico City, State of Mexico, Puebla, and Tlaxcala, had the highest prevalence of low and high myopia. Mild, moderate, and severe astigmatism significantly increased the myopia risk: 3-, 8-, and 11-fold for low myopia and 5-, 33-, and 100-fold for high myopia (all *p* < 0.001). Male sex, diabetes, and high blood pressure were associated with a lower risk (all *p* < 0.001).

**Conclusion:**

Astigmatism was a risk factor for myopia, with increasing severity raising the risk of developing both forms of myopia. Although high myopia remains relatively uncommon nationally, its increasing prevalence from childhood to early adulthood highlights the need for early detection and close monitoring to mitigate future visual impairment in Mexico.

## 1 Introduction

Refractive errors are the leading cause of visual impairment and blindness, affecting more than 123.7 million individuals worldwide (1). Myopia, also known as nearsightedness, is a refractive error characterized by the inability of the eye to focus on distant objects clearly, resulting in blurred distance vision, whereas near vision remains unaffected. This is typically caused by excessive elongation of the axial length of the eye, which prevents light from being focused directly on the retina (2). Myopia is a major contributor to distance vision impairment across all age groups, particularly in children and young adults (3). However, its impact extends beyond mere visual impairment, affecting the educational, occupational, social, and psychological wellbeing of individuals. Studies have highlighted the associations between myopia and poorer academic or work performance, reduced social interaction, and a decline in the overall quality of life (4, 5).

Both genetic predisposition and environmental exposure substantially contribute to the onset and progression of myopia (6, 7). Among the environmental determinants, increased time spent indoors, prolonged engagement in near-vision tasks, and extensive use of electronic devices are recognized as leading risk factors (7). Furthermore, some studies have associated astigmatism, a refractive condition characterized by rays of light focusing on two orthogonal focal lines rather than a single focal point, resulting in blurred vision, as a potential factor contributing to the development of myopia (8–11). According to animal studies, uncorrected astigmatism blur may disrupt the normal emmetropization process, leading to excessive axial growth, thereby influencing the onset and progression of myopia (12–14).

In a meta-analysis by Hashemi et al. (15), myopia was estimated to affect 11.7% of children and 26.5% of the global adult population. With its prevalence increasing rapidly, particularly among younger age groups, myopia has become a significant global health concern (16). This is particularly evident in East and Southeast Asian countries, where the prevalence rates among younger populations exceed 80% (17–23). The growing burden of myopia is accompanied by a rise in high myopia, a higher degree form defined by a spherical equivalent refraction (SER) of less than −6.00 diopters (D) or an axial length greater than 26 mm, which significantly elevates the risk of developing sight-threatening ocular complications, such as cataracts, glaucoma, retinal detachment, and myopic macular degeneration, potentially leading to irreversible vision loss and blindness (24, 25). If current trends continue, by 2050, myopia and high myopia will affect approximately 4,800 million and 938 million individuals worldwide, respectively (26).

At present, several scattered studies have investigated the prevalence of refractive errors in Mexico, reporting myopia rates ranging from 4.6 to 45.21%, depending on the population and geographical region studied (27–35). Nonetheless, most of these studies were conducted prior to the COVID-19 pandemic, focused predominantly on school-based populations, and were limited to local or regional settings, reducing their applicability in understanding the broader national context. Moreover, data on the prevalence of high myopia in Mexico are scarce. Existing reports are based on localized findings, making it difficult to assess the broader magnitude and public health impact of high myopia at the national level (30, 31, 34, 35). Consequently, large-scale epidemiological investigations are needed to address the knowledge gaps regarding myopia prevalence and severity on a national scale.

In this regard, Salud Digna, a private, not-for-profit, and non-governmental primary healthcare institution with a nationwide presence in Mexico, offers a wide array of diagnostic services at the primary care level, including comprehensive optometry care for the general population. Its extensive network of clinics across the country improves access to eye care services and enables the collection of valuable eye health data, helping fill knowledge gaps and support public health initiatives to reduce visual impairment nationwide. Therefore, we conducted a large-scale, multicenter study aimed at estimating the prevalence and distribution of low myopia and high myopia in a nationally representative cohort of Mexican outpatients. The findings of this study represent a comprehensive and up-to-date resource for myopia epidemiology in Mexico, providing a theoretical basis for further research and direction for the development of myopia interventions to reduce the national burden of this condition.

## 2 Methods

### 2.1 Study design and population

This retrospective, cross-sectional, clinic-based study was based on anonymized electronic health records (EHRs) retrieved from outpatients who underwent routine eye examinations at 208 Salud Digna clinics between January and December 2023. As part of the standardized ocular evaluation protocol at all clinics, certified optometrists conducted a series of assessments, including pupillary and interpupillary distance measurements, distance and near visual acuity tests, non-cycloplegic autorefraction plus subjective refinement, and documented clinical history information in a proprietary electronic clinical form as part of the routine practice. Recorded variables included demographic data (age, sex, and state of residence), eye care behavior (prior eye examinations, eyeglasses usage, and previous ophthalmologist consultation) clinical history (self-reported chronic conditions, such as diabetes or high blood pressure), and eye-related medical background (ocular surgery, trauma, and diagnosed ocular condition, including glaucoma, diabetic retinopathy, hypertensive retinopathy, or age-related macular degeneration).

A convenience sampling method was used in this study to select participants. All outpatients aged 6–100 years were initially considered for inclusion. For individuals with multiple examinations, only the most recent encounter in 2023 was considered to avoid duplication and ensure the consistency of the data. Records were excluded if they presented incomplete data or if the individual had a history of ocular surgery, ocular trauma, or ocular pathologies, including cataracts, glaucoma, diabetic retinopathy, hypertensive retinopathy, or age-related macular degeneration.

For analytical purposes, individuals were stratified into the following age groups: ≤ 10, 11–20, 21–30, 31–40, 41–50, 51–60, 61–70, 71–80, > 80 years. The final dataset incorporated demographic (age, sex, and state of residence), clinical background (self-reported diabetes or high blood pressure), eye care behavior (prior eye examinations, eyeglasses usage, and previous ophthalmologist consultation), and refractive error (sphere, cylinder, and axis) data. Given the strong correlation between the left and right eyes in spherical and cylindrical power (Spearman’s correlation coefficients: *ρ* = 0.94 and *ρ* = 0.83, respectively), only right-eye data were considered for subsequent analyses.

### 2.2 Definition of myopia

Spherical equivalent refraction (SER) was calculated by adding the sphere power to one-half of the cylinder power. Myopia was defined as a SER of ≤ − 0.50 diopters (D). Furthermore, in line with the International Myopia Institute (IMI), myopia severity was categorized into low and high myopia as −6.0 D < SER ≤ −0.50 D and SER ≤ −6.0 D, respectively (24).

### 2.3 Ethics statement, consent, and permissions

This study was approved by the Ethical Review and Research Board of Salud Digna (SDI-202505) and adhered to the approved guidelines for clinical management information, the Declaration of Helsinki, and the national regulations of Mexico (Federal Law on Personal Data Protection). Consent for using information from health records was obtained in accordance with the Mexican Federal Law on Personal Data Protection Held by Private Parties (LFPDPPP, by its Spanish acronym; https://www.diputados.gob.mx/LeyesBiblio/pdf/LFPDPPP.pdf).

Individuals who attend Salud Digna clinics accept our privacy policy, which includes the use of their information for scientific research purposes. By accepting this policy, we did not require specific informed consent from each person included in this study, as this study was a cross-sectional analysis of an electronic health registry. We adhered to the data protection and privacy regulations established by the national laws and guidelines in Mexico. The data were anonymized prior to analysis by assigning a unique ID code to protect the individuals’ identities and prevent data duplication in subsequent analyses.

### 2.4 Statistical analysis

All statistical analyses were conducted using Microsoft Excel (v.2705) and the open-source R Statistical Software (v.4.4.2; R Core Team 2023) (36). The normality of all continuous data was evaluated using the Anderson-Darling test. As the data were not normally distributed, non-parametric statistical tests were applied. Categorical variables were expressed as frequency counts and percentages, whereas continuous variables were reported as medians with interquartile ranges (IQRs). Sex-based differences among myopic individuals were compared using the chi-square test for categorical variables and the Mann–Whitney *U* test for continuous variables.

Prevalence estimates for total myopia and low and high myopia levels were reported as percentages with 95% confidence intervals (CIs) calculated using the Clopper–Pearson exact method. The association between sex and the presence or severity of myopia was evaluated using the chi-square test of independence. Additionally, to account for differences in age distribution, overall and stratified prevalence estimates by sex and state were age-adjusted using direct standardization with the World Health Organization (WHO) standard world population (37). Significant sex-related differences in the age-adjusted estimates were assessed using the *Z*-test.

To examine the influence of astigmatism severity and eye care behavior on myopia severity, two categorical predictor variables were defined. Astigmatism was defined as a minus cylinder power (Cyl) ≤ −0.75 D and classified into three levels: mild (−1.50 D < Cyl ≤ −0.75 D), moderate (−2.50 D ≤ Cyl ≤ −1.50 D), and severe (Cyl < −2.50 D) (38). Eye care behavior was condensed into a four-level categorical variable: 1) no prior eye examination and no eyeglasses use; 2) prior eye examination and no eyeglasses use; 3) no prior eye examination and eyeglasses use; and 4) prior eye examination and eyeglasses use.

Multinomial logistic regression analyses were performed to assess the association with myopia severity, which was treated as a dependent variable and categorized into three levels: no myopia (reference), low myopia, and high myopia. Simple and multiple models were fitted, including age, sex, state of residence, astigmatism severity, eye care behavior, and comorbidities as covariates. Age was treated as a continuous variable in the analysis. A stepwise selection approach was used to identify the significant predictors. Adjusted relative risk ratios (RRRs) with corresponding 95% CIs were estimated from the final multivariable model. A two-sided *p*-value < 0.05 was considered statistically significant in all analyses.

## 3 Results

### 3.1 Population characteristics

After data cleaning, a final dataset comprising 3,507,826 EHRs from outpatients was used for the retrospective analysis, of which their distribution and general characteristics are described in Supplementary Table S1. A total of 1,337,526 (38.13%) individuals with a median age of 28 years (IQR, 19, 43), including 821,838 females (61.44%) and 515,688 males (38.56%), were identified as myopic. In terms of myopia severity, 1,302,469 (97.38%) individuals had low myopia, whereas 35,057 (2.62%) had high myopia. The general characteristics of the myopic subgroup, including distributions by age, refractive error data, severity of myopia and astigmatism, eye care behavior, and comorbidities, disaggregated by sex, are presented in Table 1.

**Table 1:** Characteristics of myopic individuals among Mexican outpatients.

Characteristic	Total	Female	Male	*p*-value
	*n* = 1,337,526	*n* = 821,838	*n* = 515,688	
Age (years), median (IQR)	28 (19, 43)	29 (19, 43)	28 (18, 44)	**<0.001**^**a**^
Refractive error data (D), median (IQR)				
Sphere power (Sph)	−0.75 (−1.75, −0.25)	−0.75 (−1.75, −0.25)	−0.75 (−1.75, 0.00)	**<0.001**^**a**^
Cylinder power (Cyl)	−1.00 (−2.00, −0.50)	−1.00 (−1.75, −0.50)	−1.25 (−2.25, −0.50)	**<0.001**^**a**^
Spherical equivalent refraction (SER)	−1.38 (−2.50, −0.75)	−1.38 (−2.38, −0.75)	−1.50 (−2.50, −0.88)	**<0.001**^**a**^
Myopia severity, *n* (%)				
Low myopia (−6.0 D < SER ≤ −0.50 D)	1,302,469 (97.38)	799,296 (97.26)	503,173 (97.57)	**<0.001**^**b**^
High myopia (SER ≤ −6.0 D)	35,057 (2.62)	22,542 (2.74)	12,515 (2.43)	
Cylindrical refraction (Cyl ≤ − 0.75 D), *n* (%)				
No	462,556 (34.58)	314,259 (38.24)	148,297 (28.76)	**<0.001**^**b**^
Yes	874,970 (65.42)	507,579 (61.76)	367,391 (71.24)	
Astigmatism severity, *n* (%)				
No astigmatism	462,556 (34.58)	314,259 (38.24)	148,297 (28.76)	**<0.001**^**b**^
Mild (−1.50 D < Cyl ≤ −0.75 D)	366,952 (27.44)	228,444 (27.80)	138,508 (26.86)	
Moderate (−2.50 D ≤ Cyl ≤ −1.50 D)	305,213 (22.82)	174,367 (21.22)	130,846 (25.37)	
Severe (Cyl < −2.50 D)	202,805 (15.16)	104,768 (12.75)	98,037 (19.01)	
Eye care behavior, *n* (%)				
*Prior eye examination*				
No	608,432 (45.49)	369,541 (44.97)	238,891 (46.32)	**<0.001**^**b**^
Yes	729,094 (54.51)	452,297 (55.03)	276,797 (53.68)	
*Eyeglasses usage*				
No	569,392 (42.57)	344,784 (41.95)	224,608 (43.58)	**<0.001**^**b**^
Yes	768,134 (57.43)	477,054 (58.05)	291,080 (56.44)	
*Previous ophthalmologist consultation*				
No	1,324,282 (99.01)	813,649 (99.01)	510,633 (99.02)	0.357^b^
Yes	13,205 (0.99)	8,165 (0.99)	5,040 (0.98)	
Missing data	39 (0)	24 (0)	15 (0)	
Comorbidity, *n* (%)				
*Diabetes*				
No	1,267,524 (94.77)	780,514 (94.97)	487,010 (94.44)	**<0.001**^**b**^
Yes	70,002 (5.23)	41,324 (5.03)	28,678 (5.56)	
*High blood pressure*				
No	1,257,514 (94.02)	773,002 (94.06)	484,512 (93.95)	**0.014**^**b**^
Yes	80,012 (5.98)	48,836 (5.94)	31,176 (6.05)	

a= Mann-Whitney test U test. A *p*-value < 0.05 was considered statistically significant.b= Chi-square test. A *p*-value < 0.05 was considered statistically significant.

Regarding refractive error measurements, the median values for spherical, cylindrical, and SER were −0.75 D (IQR, −1.75, −0.25), −1.00 D (IQR, −2.00, −0.50), and −1.38 D (IQR, −2.50, −0.75), respectively. Statistically significant differences were observed between the sexes across all three parameters (all *p* < 0.001), which were greater in males. Among the myopic subgroup, 27.44, 22.82, and 15.16% concurrently exhibited mild, moderate, or severe astigmatism, respectively. Notably, 45.49% had never undergone an eye examination, 42.57% did not use eyeglasses, and 5.23 and 5.98% self-reported having diabetes and high blood pressure, respectively (Table 1).

### 3.2 Prevalence of myopia

The age-standardized prevalence of total myopia was 44.44% (95% CI, 44.36–44.52%), with a higher prevalence rate in males (45.58%; 95% CI, 45.45–45.71%) than in females (43.79%; 95% CI, 43.69–43.89%). This sex difference was statistically significant (Z = 21.4, *p* < 0.001). Regarding myopia severity, the age-standardized prevalence rates for low and high myopia were 43.31% (95% CI, 43.24–43.39%) and 1.12% (95% CI, 1.11–1.14%), respectively. Low and high myopia differed significantly between sexes. Low myopia was higher among males than females (44.49%; 95% CI, 44.36–44.62% vs. 42.65%; 95% CI, 42.55–42.75%; *Z* = 21.99, *p* < 0.001), whereas high myopia was higher in females than males (1.14; 95% CI, 1.13–1.16% vs. 1.09; 95% CI, 1.07–1.11%; *Z* = 3.92, *p* < 0.001).

### 3.3 Prevalence of myopia across age groups

**Figure 1:**
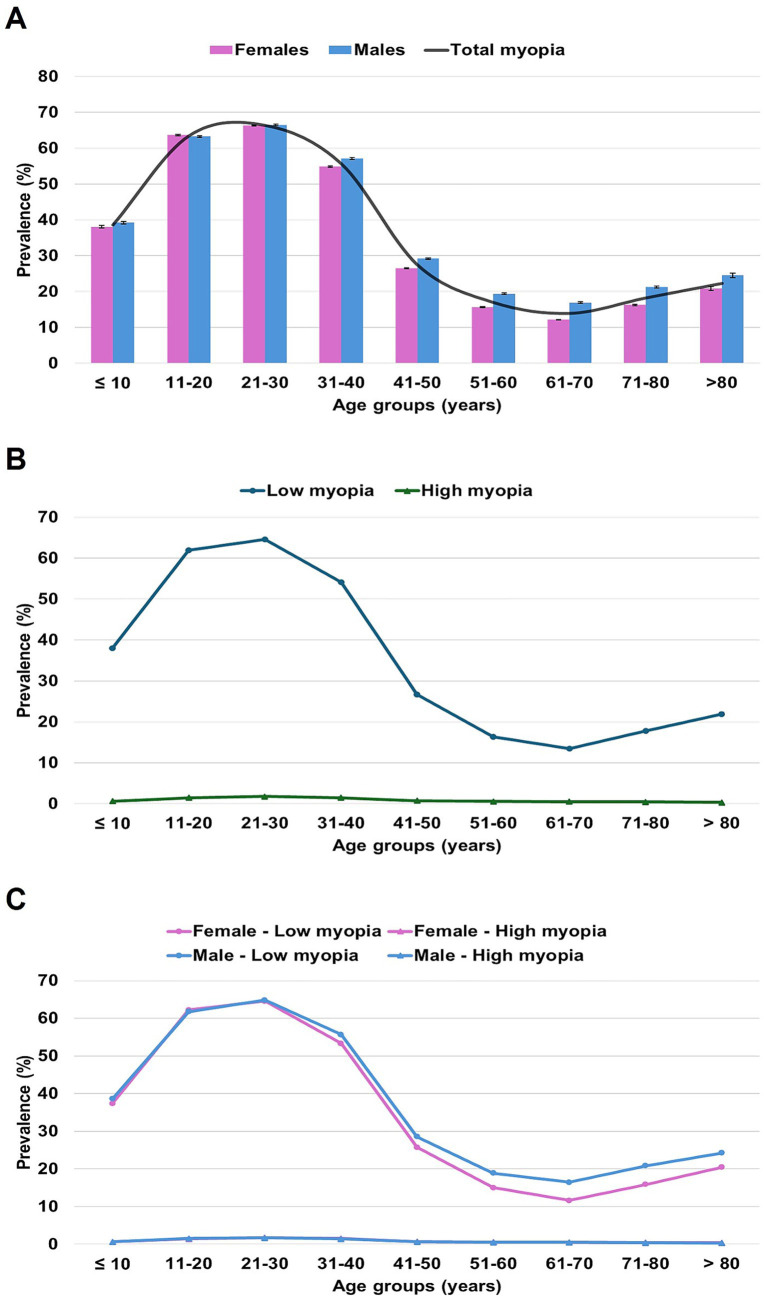
Crude prevalence of myopia among Mexican outpatients stratified by age. **(A)** Sex-specific prevalence of total myopia; **(B)** prevalence of low and high myopia; **(C)** sex-specific prevalence of low and high myopia. Bar charts display crude prevalence estimates of total myopia, with error bars representing the 95% confidence intervals. Line charts display age-related trends for total, low, and high myopia across age groups, respectively.

When analyzed by age group, the prevalence of myopia followed an *N*-shaped tendency, depicting an age-related variation in both onset and persistence throughout the lifespan (Figure 1A). The crude prevalence estimates of myopia, stratified by age and sex, are shown in Table 2.

**Table 2:** Age group- and sex-specific crude prevalence estimates of myopia among Mexican outpatients.

Age groups (years)	Total individuals (*n*)	Myopia (SER ≤ − 0.50 D)									
		General		Female			Male			*χ^2^*	*p*-value
		*n*	% (95% CI)	Total	*n*	% (95% CI)	Total	*n*	% (95% CI)		
≤ 10	152,873	59,013	38.6 (38.36–38.85)	80,165	30,496	38.04 (37.71–38.38)	72,708	28,517	39.22 (38.87–39.58)	22.39	**<0.001**
11–20	529,524	336,234	63.5 (63.37–63.63)	316,665	201,542	63.65 (63.48–63.81)	212,859	134,692	63.28 (63.07–63.48)	7.42	**0.006**
21–30	505,481	335,625	66.4 (66.27–66.53)	322,449	213,935	66.35 (66.18–66.51)	183,032	121,690	66.49 (66.27–66.7)	1.01	0.316
31–40	396,880	220,867	55.65 (55.5–55.81)	257,768	141,451	54.88 (54.68–55.07)	139,112	79,416	57.09 (56.83–57.35)	179.20	**<0.001**
41–50	651,278	178,629	27.43 (27.32–27.54)	431,257	114,373	26.52 (26.39–26.65)	220,021	64,256	29.2 (29.01–29.39)	527.14	**<0.001**
51–60	640,683	108,557	16.94 (16.85–17.04)	418,736	65,515	15.65 (15.54–15.76)	221,947	43,042	19.39 (19.23–19.56)	1447.21	**<0.001**
61–70	418,901	58,272	13.91 (13.81–14.02)	265,469	32,267	12.15 (12.03–12.28)	153,432	26,005	16.95 (16.76–17.14)	1866.15	**<0.001**
71–80	171,345	31,222	18.22 (18.04–18.41)	104,763	17,069	16.29 (16.07–16.52)	66,582	14,153	21.26 (20.95–21.57)	673.05	**<0.001**
>80	40,861	9,107	22.29 (21.88–22.69)	24,881	5,190	20.86 (20.36–21.37)	15,980	3,917	24.51 (23.85–25.19)	74.95	**<0.001**

Statistically significant values are indicated in bold.

The prevalence markedly increased from 38.6% (95% CI, 38.36–38.85%) in children under 10 years to 63.5% (95% CI, 63.37–63.63%) in adolescents aged 11–20, reaching a peak of 66.4% (95% CI, 66.27–66.53%) among young adults aged 21–30. Following this peak, prevalence declined to 55.65% (95% CI, 55.5–55.81%) in individuals aged 31–40 and dropped sharply to 27.43% (95% CI, 27.32–27.54%) in the 41–50 group; this trend continued to decrease in older adulthood, reaching 13.91% (95% CI, 13.81–14.02%) in those aged 61–70. After the 70s, a modest rebound in prevalence was observed. In most age groups, a statistically significant difference between the sexes was observed, being predominantly higher in males (*p* < 0.001) (Table 2 and Figure 1A).

In terms of severity, low myopia exhibited a similar age distribution pattern to the total myopia prevalence, whereas high myopia, although steadily decreasing with age, remained relatively constant across age groups (Figure 1B). The highest prevalence of both low and high myopia was recorded in the 21–30 age group, with rates reaching 64.65% (64.52–64.78%) for low myopia and 1.75% (95% CI, 1.71–1.79%) for high myopia. An elevated prevalence was also noted in the adjacent age groups of 11–20 and 31–40 years, underscoring early adulthood as the most affected stage (Supplementary Table S2).

Notable sex-related differences in the prevalence of low myopia emerged from the early 30s onward (Figure 1C). Although these differences were subtle, they were statistically significant across most age groups and were predominantly higher in males (*p* < 0.001). Conversely, females were more affected by high myopia across all age groups, with statistically significant differences observed in age groups from 11 to 20 through 51–60 (*p* < 0.05) (Supplementary Table S2).

### 3.4 Geographical distribution of myopia

**Figure 2:**
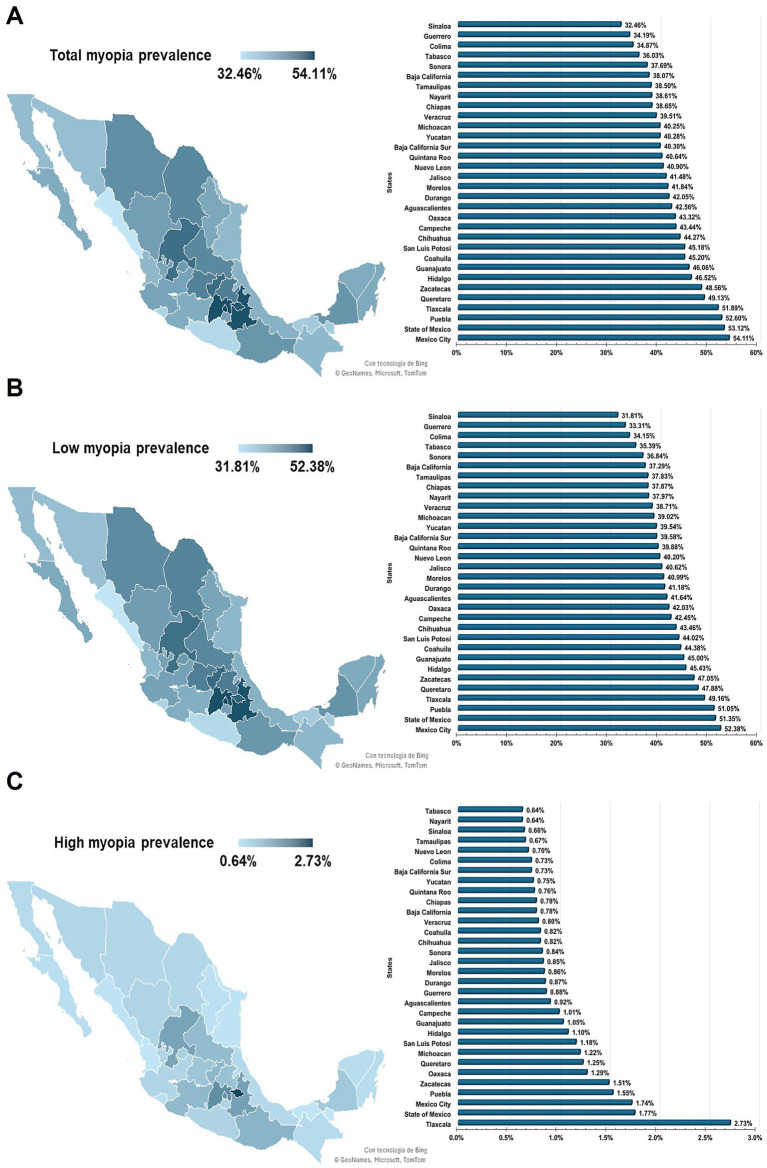
Geographic distribution of age-adjusted myopia prevalence in the Mexican outpatient cohort: **(A)** state-level prevalence of total myopia; **(B)** state-level prevalence of low myopia; **(C)** state-level prevalence of high myopia.

The spatial distribution of age-adjusted rates of total myopia was analyzed across Mexico, ranging from 32.46 to 54.11% in all the states. The highest prevalence rates were noted in Mexico City (54.11%; 95% CI, 53.85–54.38%), the State of Mexico (53.12%; 95% CI, 52.91–53.33%), Puebla (52.6%; 95% CI, 52.18–53.03%), and Tlaxcala (51.89%; 95% CI, 50.35–53.47%), whereas the lowest prevalence were in Sinaloa (32.46%; 95% CI, 32.16–32.77%), Guerrero (34.19%; 95% CI, 33.42–34.98%), Colima (34.87%; 95% CI, 34.06–35.7%), and Tabasco (36.03%; 95% CI, 35.34–36.73%) (Figure 2A). When analyzed by sex, statistically significant differences were observed in most states, with a higher prevalence among males (*p* < 0.001), although, no significant difference was found in Campeche (Supplementary Table S3).

When analyzed by severity, the geographic distribution of low myopia prevalence showed a pattern comparable to that of total myopia. Low myopia was most prevalent in Mexico City (52.38%; 95% CI, 52.12–52.64%), the State of Mexico (51.35%; 95% CI, 51.14–51.56%), Puebla (51.05%; 95% CI, 50.64–51.47%), and Tlaxcala (49.16%; 95% CI, 47.66–50.7%) (Figure 2B). Similarly, low myopia was more prevalent among males across all states, except in Campeche, where the difference between the sexes was not statistically significant (*p* = 0.573) (Supplementary Table S4).

Regarding high myopia, although the prevalence rates ranged from 0.64 to 2.73%, most states remained below 1%. The highest prevalence was observed in Tlaxcala at 2.73% (95% CI, 2.39–3.12%), followed by the State of Mexico, Mexico City, Puebla, and Zacatecas, where the rates ranged from 1.51 to 1.77% (Figure 2C). Although high myopia was predominantly prevalent among females across all states, only in five of the 32 states were statistically significant sex-related differences observed (Supplementary Table S5).

### 3.5 Factors associated with the severity of myopia

According to simple multinomial logistic regression analyses, age, sex, state of residence, severity of astigmatism, eye care behavior, diabetes, and high blood pressure were independently associated with the severity of myopia. Excluding male sex, which was not significantly associated with high myopia (RRR = 1.01; 95% CI, 0.988–1.032; *p* = 0.3904), the rest of the variables showed statistically significant associations with both low and high myopia (all *p* < 0.001) and hence were included in the multivariable model (Supplementary Table S6).

The results of the multiple multinomial logistic regression analysis are presented in Supplementary Table S7. As expected, the risk of both low and high myopia decreased significantly with each additional year of age, reflecting a continuous age-related decline in myopia prevalence. This effect was stronger for low myopia, with a 4.40% reduction in risk per year, compared with a 3.50% reduction for high myopia. Regardless of the severity of myopia, male individuals had lower risk of low (RRR = 0.952; 95% CI, 0.947–0.957; *p* < 0.001) or high myopia (RRR = 0.712, 95% CI, 0.695–0.728; *p* < 0.001) when compared to females.

Regarding the state of residence, most states had a significantly lower risk of both low and high myopia than the State of Mexico; however, relevant insights were observed for three states. Mexico City and Puebla were notable for being the only states with an approximately 8 and 9% increase in the risk of having low myopia (*p* < 0.001), respectively. In contrast, for high myopia, Puebla, Mexico City, and Tlaxcala were unique, with nearly 5, 10, and 50% increases in risk, respectively. However, in Puebla, this association was not statistically significant (*p* = 0.0636).

Astigmatism of any degree was significantly associated with an increased risk of both low and high myopia, with the risk escalating according to astigmatism severity (all *p* < 0.001). For individuals with low myopia, the presence of mild, moderate, or severe astigmatism corresponded to approximately 3-, 8-, and 11-fold higher risks, respectively, than in those without astigmatism. The association was substantially stronger in individuals with high myopia, where mild, moderate, and severe astigmatism were linked to 5-, 33-, and 100-fold increased risks, respectively.

Distinct associations were observed between eye care behavior and myopia severity. Individuals with prior eye examination but no eyeglasses use had a slightly increased risk of low myopia (RRR = 1.033; 95% CI, 1.014–1.052; *p* < 0.001) but a significantly reduced risk of high myopia (RRR = 0.741; 95% CI, 0.655–0.839; *p* < 0.001) compared with those without eye examination and no eyeglasses use. Individuals without prior examination but using eyeglasses showed markedly and significantly increased risks of low (RRR = 1.611; 95% CI, 1.591–1.632) and high myopia (RRR = 3.026; 95% CI, 2.88–3.179). Finally, individuals with prior eye examinations and eyeglass use had the highest risk of myopia severity, with RRRs of 1.835 (95% CI, 1.824–1.845) for low myopia and 3.239 (95% CI, 3.157–3.324) for high myopia.

Diabetes and high blood pressure were negatively associated with the presence of both low and high myopia (both *p* < 0.01). Individuals with diabetes had a reduced risk of having low myopia (RRR = 0.985; 95% CI, 0.974–0.995; *p* = 0.0043) or high myopia (RRR = 0.919; 95% CI, 0.874–0.966; *p* < 0.001) than those without diabetes. Similarly, individuals with high blood pressure had a lower risk of having low myopia (RRR = 0.793; 95% CI, 0.786–0.801; *p* < 0.001) or high myopia (RRR = 0.708; 95% CI, 0.675–0.743; *p* < 0.001).

## 4 Discussion

This large-scale, nationwide, retrospective study provides one of the most comprehensive and current data on the prevalence, severity, and geographic distribution of myopia in the Mexican population, with specific attention to variations by age and sex. Based on data from over 3.50 million outpatients aged 6–100 years, our analyses provide five key observations: 1) the overall crude prevalence of myopia was 38.13%, with 37.13% classified as having low myopia and 1.00% as having high myopia; 2) children, adolescents, and young adults represented the most affected age strata; 3) low myopia predominated among males, whereas females exhibited a relatively higher prevalence of high myopia; 4) while myopia was prevalent across all states of Mexico, both forms of myopia were most concentrated in states within the Central region, specifically in Mexico City, State of Mexico, Puebla, and Tlaxcala; and 5) astigmatism significantly increased the risk of both low and high myopia in a severity-dependent manner.

These findings represent a significant update to the epidemiological understanding of myopia in Mexico, expanding the scope and relevance of previous studies, most of which are limited by demographic constraints and nearly all of which predate the COVID-19 pandemic (27–35). It is noteworthy that this study was framed within the post-COVID period to situate our results in the current epidemiological context. However, it was not designed to assess the changes attributable to the pandemic. Direct comparisons between pre- and post-pandemic myopia patterns were not feasible because of the absence of pre-pandemic baseline or longitudinal data in our dataset. Consequently, causal or temporal inferences regarding the pandemic-related changes in myopia cannot be made. Future longitudinal and population-based studies specifically designed to compare pre- and post-pandemic data are needed to quantify the long-term impact of the COVID-19 pandemic on myopia prevalence and severity in the Mexican population. Nevertheless, our findings provide valuable insights for informing both national eye health strategies and global efforts to control the growing myopia burden.

The variation in myopia prevalence across global regions is well-established, with East and Southeast Asian populations consistently reporting the highest myopia rates, often exceeding 80% among younger populations (15, 20, 39, 40). Although the available data from the Americas has indicated lower prevalence, recent projections suggest a steady increase through the region. According to Holden et al., the projected myopia prevalence for 2030 is estimated to be 48.5% in North America, 41.6% in Central Latin America, 40.7% in Southern America, and 35.9% in Tropical Latin America (26).

We estimated an overall age-standardized rate for total myopia of 44.44% (95% CI, 44.36–44.52%), placing Mexico within these projected trajectories, particularly aligning with trends observed in Central and North America. This estimation is also significantly higher than the 15.9% pooled rate reported in the most recent systematic review and meta-analysis of myopia in the Americas, which considered studies with varying age groups, methodologies, and prevalence rates (1.2–48%) (41). However, the representativeness of this pooled prevalence may be limited, as it only included data from the U.S. and Brazilian populations.

When subclassified by severity, we estimated age-standardized prevalence rates of 43.31% (95% CI, 43.24–43.39%) and 1.12% (95% CI, 1.11–1.14%) for low and high myopia, respectively. Although these estimates are lower than those reported in East and Southeast Asian countries, they still represent a significant public health burden, considering that two of five individuals face the potential risk of progressing to high myopia and its associated sight-threatening complications (42). This risk is particularly concerning among individuals aged ≤ 10, 11–20, 21–30, and 31–40 years, where the highest crude prevalences of total, low, and high myopia were consistently observed.

Several epidemiological studies have investigated the prevalence of refractive errors in the Mexican population, revealing considerable variability in reported myopia rates, ranging from 4.6 to 45.21%, depending on the region and the study population (27–35). However, it is important to highlight that most existing reports have focused predominantly on school-based populations, and nearly all were conducted at the local or regional level, thereby limiting their generalizability to the broader Mexican population. Only three studies have included broader age ranges beyond school-aged children, offering more inclusive, but still limited, perspectives on the national prevalence of this refractive condition (28, 31, 32).

Compared with these earlier reports, our overall estimate exceeded the previously reported rate by Gomez-Salazar et al., who found a myopia prevalence of 24.8% among outpatients aged 6–90 years old from 14 states across Mexico (28). Similarly, Barba-Gallardo et al. reported significantly lower rates: 7.1% in individuals under 18 years of age and 8.6% in adults over 18 years of age, based on data from Aguascalientes (32). In contrast, our estimate was slightly lower than that reported by Milán-Castilo et al. (31), who reported a prevalence of 45.21% in adults aged 21–89 years residing in Mexico City.

While the higher prevalence reported in our study may reflect recent increases in myopia rates compared to early findings, several factors should be considered. Demographic differences, including age distribution, sex, urban vs. rural residence, and geographic region, play significant roles. Moreover, methodological inconsistencies across studies, such as variations in sampling strategies, sample sizes, and types of ocular examinations performed, may also contribute to the discrepancies in the findings (43). For example, we reported age-adjusted prevalence estimates to reduce age-related biases, whereas previous studies did not apply age-standardization techniques. This lack of standardization can lead to potential biases because of differences in the age composition of the assessed population groups, making direct comparisons inappropriate and warranting cautious interpretation of the results.

In relation to age-specific prevalence, we observed a higher crude myopia prevalence of 38.6, 63.5, 66.4, and 55.65% among individuals aged ≤ 10, 11–20, 21–30, and 31–40 years, respectively. These prevalence rates exceed those reported by Gomez-Salazar et al. and Barba-Gallardo et al. within similar age strata (28, 32). However, all studies consistently showed that children, adolescents, and young adults were the groups most affected by myopia. This age-related burden can partly be attributed to environmental and behavioral factors, such as increased near work and reduced time spent outdoors due to schooling in children and adolescents, and occupational demands during the productive years of young adults (7, 44). Hence, to prevent academic performance declines among students and productivity losses among working-age individuals, it is essential to strengthen public health policies focused on prevention, education, and expanded access to vision care services across the country.

In a global context, the projected prevalence of high myopia by 2030 is estimated to be 6.1% (26). While the prevalence of high myopia remains low in European populations (0.8%) (45), other countries show much higher rates of high myopia. For example, in the United States (46), high myopia prevalence has reached approximately 8.3%, and in China, it ranges from 9.5 to 18.2% (40, 47). Data on the epidemiology of high myopia in Mexico are severely limited, and existing studies indicate a low prevalence.

We estimated an overall age-adjusted prevalence of high myopia at 1.12% (95% CI, 1.11–1.14%), offering one of the first large-scale national benchmarks for this vision-threatening condition. This estimate is significantly lower than the 5.79% reported by Milán-Castillo et al. (31). This difference was more evident when analyzed by age groups, since our prevalence estimates for individuals over 40 years ranged from 0.72 to 0.36%, decreasing in a linear manner. On the contrary, the prevalence of high myopia increased with age up to the fourth decade of life, with rates of 0.64, 1.49, 1.75, and 1.5% among individuals aged ≤ 10, 11–20, 21–30, and 31–40 years, respectively. These patterns were consistent with earlier findings of Teran et al. (30, 34, 35), who observed age-specific prevalence rates of 1.24% for elementary school children, 0.4% for middle school students, and 2.36% for high school students from Sinaloa.

Although our findings suggest that high myopia remains relatively uncommon in Mexico at the national level, the steady increase in prevalence from childhood to early adulthood highlights the need for close monitoring of myopia progression, especially in younger populations. Given the known risk of vision-threatening complications associated with high myopia, including cataracts, peripheral retinal degeneration and retinal detachment, myopic maculopathy, and open-angle glaucoma, even a modest prevalence at the population level may have significant public health implications over time (25). This underscores the importance of integrating high myopia surveillance into national vision screening programs and considering early intervention strategies to reduce disease progression in at-risk individuals with high myopia.

Most literature from other countries or regions suggests that myopia prevalence is higher among females (17–19, 30, 32, 33, 35, 46, 48–52). We found sex-specific differences in the prevalence of total myopia. Overall, males had a significantly higher rate of myopia (45.58%; 95% CI, 45.45–45.71%) compared to females (43.79%; 95% CI, 43.69–43.89%), a finding that aligns with previous studies conducted among Mexican schoolchildren and the general population (28, 34). However, adjusted multinomial logistic regression analysis indicated that males had lower risk of both low and high myopia.

While the sex-related pattern in the prevalence of low myopia was generally similar to that of total myopia, a subtle downward trend in prevalence among females emerged from the early 30s onward. Recently, it was observed that myopia progression tends to be faster among female individuals (53). In our study, females exhibited a relatively higher prevalence of high myopia than male regardless of age strata, a pattern consistent with earlier research (54).

These sex-specific differences may reflect underlying biological and hormonal factors, such as higher levels of sex hormones, specifically follicle stimulating hormone and luteinizing hormone, which could influence myopia onset and progression in females (55). Additionally, extracellular matrix remodeling and collagen fiber synthesis in highly myopic corneas are more active among females, suggesting a greater susceptibility to myopia progression and severity (56). Behavioral and environmental factors also play a role, as females may spend more time on near work activities such as reading, writing, and using electronic devices, while spending less time outdoors, increasing their risk of myopia development (7, 44). However, these factors could not be directly evaluated in the present analysis because of the dependance on routinely collected clinical data. This highlights the importance of adapting myopia management strategies accounting for sex-specific biological, behavioral, environmental, and lifestyle factors into a comprehensive framework to enhance the effectiveness of current interventions.

Regarding geographic distribution, our results showed that both low and high myopia are widespread nationally. However, there was a notable concentration in the Central region, specifically in Mexico City, State of Mexico, Puebla, and Tlaxcala, where age-standardized prevalence estimates for low myopia were the highest: 52.38% (95% CI, 52.12–52.64%), 51.35% (95% CI, 51.14–51.56%), 51.05% (95% CI, 50.64–51.47%), and 49.16% (95% CI, 47.66–50.7%), respectively. In the case of high myopia, the highest prevalence was similarly concentrated in Tlaxcala (2.73%; 95% CI, 2.39–3.12%), State of Mexico (1.77%; 95% CI, 1.73–1.81%), Mexico City (1.74%; 95% CI, 1.69–1.78%), and Puebla (1.55%; 95% CI, 1.48–1.62%).

These prevalence estimates are significantly higher than those previously reported by Gomez-Salazar et al. (28), who reported overall myopia prevalence of 36% in Mexico City, 35.6% in Puebla, and 31% in the State of Mexico. Such differences may suggest a potential increase in the burden of myopia over time in these densely populated areas, likely influenced by urban-related risk factors, such as reduced time spent outdoors, increased exposure to near work, and lifestyle changes associated with urbanization. Likewise, improved access to diagnostic services in these states may partially explain the higher detection rates; however, this does not entirely account for the magnitude of the observed differences.

Furthermore, the results above were further supported by multinomial logistic regression analysis, which indicated that these states were significantly associated with an increased risk of developing both forms of myopia after confounding adjustment. Individuals from Mexico City and Puebla had 8 and 9% higher risks, respectively, of developing low myopia compared to those in the State of Mexico, which had the highest overall prevalence, whereas the risk for high myopia was 10% higher in Mexico City and markedly 50% higher in Tlaxcala. However, because information enabling stratification by urban–rural setting and socioeconomic status was not systematically collected, the assessment of potential geographic and social disparities was limited in this study. Despite this limitation, these findings highlight the importance of strengthening regional strategies into urban populations and particularly, the elevated risk observed in Tlaxcala for high myopia warrants targeted investigations into possible contributing factors, including genetic predisposition, environmental exposures, socioeconomic conditions, and disparities in healthcare access.

Analogously, our findings also highlight the strong and severity-dependent association between astigmatism and both forms of myopia. To our knowledge, this is the first study to describe the interrelationship between these variables in a Mexican cohort. Multinomial logistic regression indicated that the presence of astigmatism significantly increased the risk of developing both low and high myopia, with the risk rising according to astigmatism severity. Individuals with mild, moderate, and severe astigmatism had a 3-, 8-, and 11-fold increased risk of developing low myopia, respectively, relative to those without astigmatism, whereas the risk for high myopia considerably increased by 5-, 33-, and 100-fold, respectively.

These findings suggest that astigmatism may not only coexist with myopia but may also contribute to its onset or progression, particularly in more severe cases. A recent longitudinal study by Liang et al. explored the influence of astigmatism and its axis orientation on axial elongation in Chinese schoolchildren. The study found that high-magnitude astigmatism, particularly with against-the-rule (ATR) or oblique orientation, exerted the fastest rates of axial length growth in both myopic compound and emmetropic eyes, possibly caused by horizontally or diagonally oriented hyperopic retinal defocus, which may serve as a visual signal that triggers ocular growth and contributes to myopia development (57).

This underscores the critical role of astigmatism in influencing ocular growth and impairing normal refractive development towards myopia. Early detection, timely correction, and close monitoring of moderate to high astigmatism, particularly ATR and oblique orientations, may help identify children at greater risk and diminish myopia progression, allowing for more proactive management. Furthermore, longitudinal prospective research in the Mexican population is needed to confirm these associations and explore the underlying mechanisms using ocular biometric data, such as axial length, corneal curvature, and anterior segment parameters. Understanding whether these biomechanical interactions are consistent across different demographic and environmental contexts in the country will be key to developing enhanced population-specific interventions.

Our study has several strengths. First, the large sample size of over 3.50 million individuals aged 6–100 years across all Mexican states provided robust statistical power and nationwide representation, which allowed us to perform in-depth age- and sex-specific prevalence estimations of myopia and high myopia in Mexico. Second, the use of age-standardized prevalence rates accounted for potential biases related to the population’s age structure, strengthens the validity of the findings. Third, by analyzing data from 2023, this study represents a significant update to the epidemiological understanding of myopia in Mexico, generating the most recent and valuable resource within a post-pandemic context.

However, several limitations should be acknowledged. First, the use of non-cycloplegic refraction data may have overestimated the prevalence of myopia, particularly in younger individuals owing to active accommodative effects. Because of the nationwide multicenter setting, it is difficult to apply cycloplegic refraction in each captured individual and therefore our findings should be interpreted with caution, particularly when comparing prevalence estimates with studies employing cycloplegic protocols. However, given the large sample size, the trend of increased prevalence in younger age strata remains a valuable indicator of the underlying epidemiological patterns.

Second, the retrospective and cross-sectional design cannot establish causal relationships between the prevalence of low and high myopia and sex-specific differences, as well as the observed association between astigmatism severity and both forms of myopia. Therefore, further longitudinal prospective studies are needed, especially those incorporating ocular biometric data, to determine the temporal and mechanistic relationships between astigmatism and myopia development and progression.

Third, the exclusive use of electronic clinic-based data introduces potential selection bias, limiting the generalizability of the findings to a broader population sample. Finally, data on key behavioral and environmental factors, such as socioeconomic status, educational attainment, city-level urban–rural classification, outdoor exposure, and screen time, were not available in routine clinical documentation. The absence of these variables prevented a more detailed assessment of the lifestyle and environmental determinants of myopia, highlighting the need for future studies incorporating these measures.

Despite these limitations, this study underscores the growing burden of myopia in Mexico, establishes the first national epidemiological precedent of high myopia, and provides valuable insights to inform public health planning and lays the foundation for future clinical and epidemiological research in the field of visual care.

## 5 Conclusion

This retrospective cross-sectional study found a moderate overall prevalence of total myopia among the Mexican outpatient cohort, with children, adolescents, and young adults being the most affected groups by both low and high myopia. Regardless of age strata, low myopia was more prevalent among males, whereas females exhibited a relatively higher prevalence of high myopia. Astigmatism was a risk factor for myopia, and increasing severity grades increased the risk of developing and progressing of both low and high myopia. Although our findings suggest that high myopia remains relatively uncommon in Mexico at the national level, the steady increase in its prevalence from childhood to early adulthood highlights the need for close monitoring of myopia progression, especially in younger populations.

A coordinated action from the public and private healthcare sectors is needed to strengthen the early detection and longitudinal surveillance of myopia progression, as well as to promote timely therapeutic engagement nationwide. Expanding the accessibility and affordability of proven optical interventions for myopia management, such as peripheral defocus-based lenses or diffusion optics technology eyeglasses, will be essential to curb progression and reduce future visual impairment in at-risk populations, namely, children and adolescents.

## Data Availability

The data analyzed in this study is subject to the following licenses/restrictions: The datasets presented in this article are not readily available because restrictions established by the Mexican Federal Law on the Protection of Personal Data. Requests to access these datasets should be directed to Jonathan Alcántar-Fernández (jonathan.alcantar@salud-digna.org).
