# Supplementary material for: Prevalence and distribution of low and high myopia in Mexican outpatients: a nationwide cross-sectional clinic-based study
Source: Front Med (Lausanne). 2026 Feb 23;13:1753396. doi: 10.3389/fmed.2026.1753396 (PMC12967929; doi:10.3389/fmed.2026.1753396)
Supplement: Supplementary file 1 [file Data_Sheet_1.PDF]

Supplementary information

Table S1. General characteristics of Mexican outpatients stratified by sex

Characteristic	Total	Female	Male	*p*-value
	n = 3,507,826	*n* = 2,222,153	*n* = 1,285,673	
**Age (years), median (IQR)**	44 (24, 56)	44 (25, 56)	45 (22, 56)	**< 0.001**[a]
**Refractive error data (D), median (IQR)**				
Sphere power (Sph)	0.00 (-0.50, +1.25)	0.00 (-0.50, +1.25)	0.00 (-0.50, +1.25)	**< 0.001**[a]
Cylinder power (Cyl)	-0.50 (-1.00, 0.00)	-0.50 (-1.00, 0.00)	-0.50 (-1.25, 0.00)	**< 0.001**[a]
Spherical equivalent refraction (SER)	-0.12 (-1.00, +1.00)	0.00 (-1.00, +1.12)	-0.12 (-1.12, +0.75)	**< 0.001**[a]
**Age groups (years), *n* (%)**				
≤ 10	152,873 (4.36)	80,165 (3.61)	72,708 (5.66)	
11-20	529,524 (15.10)	316,665 (14.25)	212,859 (16.56)	
21-30	505,481 (14.41)	322,449 (14.51)	183,032 (14.24)	
31-40	396,880 (11.31)	257,768 (11.60)	139,112 (10.82)	
41-50	651,278 (18.57)	431,257 (19.41)	220,021 (17.11)	**< 0.001**[b]
51-60	640,683 (18.26)	418,736 (18.84)	221,947 (17.26)	
61-70	418,901 (11.94)	265,469 (11.95)	153,432 (11.93)	
71-80	171,345 (4.99)	104,763 (4.71)	66,582 (5.18)	
> 80	40,861 (1.16)	24,881 (1.12)	15,980 (1.24)	
**SER (D), *n* (%)**				
SER ≤ -0.50	1,337,526 (38.13)	821,838 (36.98)	515,688 (40.11)	
-0.50 < SER < 0.50	1,013,787 (28.90)	633,325 (28.50)	380,462 (29.59)	**< 0.001**[b]
SER ≥ 0.50	1,156,513 (32.97)	766,990 (34.54)	389,523 (30.30)	
**Cylindrical refraction (Cyl ≤ -0.75 D), *n* (%)**				
No	2,091,451 (59.62)	1,382,225 (62.20)	709,226 (55.16)	**< 0.001**[b]
Yes	1,416,375 (40.38)	839,928 (37.80)	576,447 (44.84)	
**Eye care behavior, *n* (%)**				
Prior eye examination				
No	1,829,344 (52.15)	1,127,867 (50.76)	701,477 (54.56)	**< 0.001**[b]
Yes	1,678,482 (47.85)	1,094,286 (49.24)	584,196 (45.44)	
Eyeglasses usage				
No	1,732,669 (49.39)	1,061,466 (47.77)	671,203 (52.21)	**< 0.001**[b]
Yes	1,775,157 (50.61)	1,160,687 (52.23)	614,470 (47.79)	
Previous ophthalmologist consultation				
No	3,466,407 (98.82)	2,195,650 (98.81)	1,270,757 (98.84)	
Yes	41,308 (1.18)	26,429 (1.19)	14,879 (1.16)	**0.021**[b]
Missing data	111 (0.00)	74 (0.00)	37 (0.00)	
**Comorbidity, *n* (%)**				
Diabetes				
No	3,198,947 (91.19)	2,023,718 (91.07)	1,175,229 (91.41)	**< 0.001**[b]
Yes	308,879 (8.81)	198,435 (8.93)	110,444 (8.59)	
High blood pressure				
No	3,105,947 (88.54)	1,952,374 (87.86)	1,153,573 (89.73)	**< 0.001**[b]
Yes	401,879 (11.46)	269,779 (12.14)	132,100 (10.27)	

D, diopters; IQR, interquartile range

[a]Mann-Whitney test U test. A *p*-value < 0.05 was considered statistically significant

[b]Chi-square test. A *p*-value < 0.05 was considered statistically significant

Supplementary information

Table S2. Age group- and sex-specific crude prevalence estimates of low and high myopia among Mexican outpatients

Age (years)	Total individuals (*n*)	Low myopia (-6.0 D < SER ≤ -0.50 D)									
		General		Female			Male			χ^2	*p*-value
		n	% (95% CI)	Total	*n*	% (95% CI)	Total	*n*	% (95% CI)		
≤ 10	152,873	58,036	37.96 (37.72–38.21)	80,165	29,977	37.39 (37.06–37.73)	72,708	28,059	38.59 (38.24–38.95)	23.20	**< 0.001**
11-20	529,524	328,319	62 (61.87–62.13)	316,665	196,904	62.18 (62.01–62.35)	212,859	131,415	61.74 (61.53–61.94)	10.58	**0.001**
21-30	505,481	326,784	64.65 (64.52–64.78)	322,449	208,155	64.55 (64.39–64.72)	183,032	118,629	64.81 (64.59–65.03)	3.42	0.064
31-40	396,880	214,908	54.15 (53.99–54.3)	257,768	137,470	53.33 (53.14–53.52)	139,112	77,438	55.67 (55.4–55.93)	198.42	**< 0.001**
41-50	651,278	173,913	26.7 (26.6–26.81)	431,257	111,136	25.77 (25.64–25.9)	220,021	62,777	28.53 (28.34–28.72)	567.86	**< 0.001**
51-60	640,683	104,863	16.37 (16.28–16.46)	418,736	63,015	15.05 (14.94–15.16)	221,947	41,848	18.85 (18.69–19.02)	1535.15	**< 0.001**
61-70	418,901	56,197	13.42 (13.31–13.52)	265,469	30,949	11.66 (11.54–11.78)	153,432	25,248	16.46 (16.27–16.64)	1926.47	**< 0.001**
71-80	171,345	30,490	17.79 (17.61–17.98)	104,763	16,600	15.85 (15.62–16.07)	66,582	13,890	20.86 (20.55–21.17)	700.26	**< 0.001**
> 80	40,861	8,959	21.93 (21.53–22.33)	24,881	5,090	20.46 (19.96–20.96)	15,980	3,869	24.21 (23.55–24.88)	80.11	**< 0.001**

Age (years)	Total individuals (*n*)	High myopia (SER ≤ -6.0 D)									
		General		Female			Male			χ^2	*p*-value
		n	% (95% CI)	Total	*n*	% (95% CI)	Total	*n*	% (95% CI)		
≤ 10	152,873	977	0.64 (0.6–0.68)	80,165	519	0.65 (0.59–0.71)	72,708	458	0.63 (0.57–0.69)	0.18	0.668
11-20	529,524	7,915	1.49 (1.46–1.53)	316,665	4,638	1.46 (1.42–1.51)	212,859	3,277	1.54 (1.49–1.59)	4.85	**0.028**
21-30	505,481	8,841	1.75 (1.71–1.79)	322,449	5,780	1.79 (1.75–1.84)	183,032	3,061	1.67 (1.61–1.73)	9.81	**0.002**
31-40	396,880	5,959	1.5 (1.46–1.54)	257,768	3,981	1.54 (1.5–1.59)	139,112	1,978	1.42 (1.36–1.49)	9.17	**0.002**
41-50	651,278	4,716	0.72 (0.7–0.75)	431,257	3,237	0.75 (0.73–0.78)	220,021	1,479	0.67 (0.64–0.71)	12.45	**< 0.001**
51-60	640,683	3,694	0.58 (0.56–0.6)	418,736	2,500	0.6 (0.57–0.62)	221,947	1,194	0.54 (0.51–0.57)	8.83	**0.003**
61-70	418,901	2,075	0.5 (0.47–0.52)	265,469	1,318	0.5 (0.47–0.52)	153,432	757	0.49 (0.46–0.53)	0.02	0.890
71-80	171,345	732	0.43 (0.4–0.46)	104,763	469	0.45 (0.41–0.49)	66,582	263	0.4 (0.35–0.45)	2.66	0.103
> 80	40,861	148	0.36 (0.31–0.43)	24,881	100	0.4 (0.33–0.49)	15,980	48	0.3 (0.22–0.4)	2.78	0.095

CI, confidence interval; SER, spherical equivalent refraction. The Chi-square test of independence was used to test the association between sex and low and high myopia, respectively. A *p*-value < 0.05 was considered statistically significant.

Supplementary information

Table S3. Crude and age-adjusted state-level prevalence estimates of myopia among Mexican outpatients

**State**	**Total individuals (*n*)**	**Myopia (SER ≤ -0.50 D)**												
		**General**			**Female**				**Male**					
		n	Crude % (95% CI)	Age-adjusted[a] % (95% CI)	Total	*n*	Crude % (95% CI)	Age-adjusted[a] % (95% CI)	Total	*n*	Crude % (95% CI)	Age-adjusted[a] % (95% CI)	χ^2	*p*-value
Aguascalientes	53,003	20,490	38.66 (38.24–39.07)	42.56 (41.96–43.17)	33,475	12,600	37.64 (37.12–38.16)	41.92 (41.16–42.7)	19,528	7,890	40.4 (39.71–41.1)	43.72 (42.73–44.72)	39.72	**< 0.001**
Baja California	237,989	80,307	33.74 (33.55–33.93)	38.07 (37.79–38.34)	147,674	48,909	33.12 (32.88–33.36)	37.33 (36.99–37.68)	90,315	31,398	34.76 (34.45–35.08)	39.22 (38.77–39.67)	67.86	**< 0.001**
Baja California Sur	13,893	4,620	33.25 (32.47–34.04)	40.3 (39.08–41.56)	8,970	2,849	31.76 (30.8–32.74)	38.62 (37.11–40.2)	4,923	1,771	35.97 (34.63–37.33)	43.3 (41.21–45.48)	25.41	**< 0.001**
Campeche	11,260	3,971	35.27 (34.38–36.16)	43.44 (42.03–44.9)	6,870	2,405	35.01 (33.88–36.15)	43.77 (41.93–45.69)	4,390	1,566	35.67 (34.25–37.11)	43.07 (40.87–45.37)	0.52	0.472
Chiapas	73,934	23,192	31.37 (31.03–31.7)	38.65 (38.12–39.19)	46,352	14,130	30.48 (30.07–30.91)	38.33 (37.65–39.03)	27,582	9,062	32.85 (32.3–33.41)	39.28 (38.43–40.15)	45.14	**< 0.001**
Chihuahua	89,474	32,772	36.63 (36.31–36.94)	44.27 (43.77–44.77)	57,123	20,412	35.73 (35.34–36.13)	43.89 (43.26–44.53)	32,351	12,360	38.21 (37.68–38.74)	44.93 (44.11–45.77)	54.40	**< 0.001**
Coahuila	73,016	28,575	39.14 (38.78–39.49)	45.2 (44.65–45.75)	47,229	18,110	38.35 (37.91–38.79)	44.74 (44.06–45.44)	25,787	10,465	40.58 (39.98–41.18)	45.97 (45.06–46.9)	35.05	**< 0.001**
Colima	29,598	7,919	26.76 (26.25–27.26)	34.87 (34.06–35.7)	18,373	4,810	26.18 (25.55–26.82)	35.02 (33.97–36.11)	11,225	3,109	27.7 (26.87–28.54)	34.72 (33.45–36.04)	8.19	**0.004**
Durango	62,126	22,111	35.59 (35.21–35.97)	42.05 (41.48–42.64)	40,713	14,088	34.6 (34.14–35.07)	41.6 (40.88–42.34)	21,413	8,023	37.47 (36.82–38.12)	42.83 (41.87–43.81)	50.24	**< 0.001**
Guanajuato	168,612	68,656	40.72 (40.48–40.95)	46.06 (45.7–46.42)	104,987	40,982	39.04 (38.74–39.33)	45.03 (44.57–45.49)	63,625	27,674	43.5 (43.11–43.88)	47.82 (47.24–48.4)	326.49	**< 0.001**
Guerrero	35,753	8,697	24.33 (23.88–24.77)	34.19 (33.42–34.98)	23,562	5,548	23.55 (23.01–24.09)	33.85 (32.87–34.86)	12,191	3,149	25.83 (25.06–26.62)	34.99 (33.7–36.32)	22.77	**< 0.001**
Hidalgo	24,334	10,211	41.96 (41.34–42.58)	46.52 (45.59–47.46)	15,102	6,176	40.9 (40.11–41.68)	46.11 (44.91–47.33)	9,232	4,035	43.71 (42.69–44.73)	47.35 (45.86–48.88)	18.60	**< 0.001**
Jalisco	248,652	89,785	36.11 (35.92–36.3)	41.48 (41.19–41.76)	157,599	55,553	35.25 (35.01–35.49)	40.91 (40.55–41.28)	91,053	34,232	37.6 (37.28–37.91)	42.4 (41.94–42.87)	137.69	**< 0.001**
Mexico City	382,552	178,794	46.74 (46.58–46.9)	54.11 (53.85–54.38)	243,069	110,129	45.31 (45.11–45.51)	53.53 (53.19–53.87)	139,483	68,665	49.23 (48.97–49.49)	55.2 (54.77–55.63)	547.22	**< 0.001**
Michoacan	70,184	23,332	33.24 (32.9–33.59)	40.25 (39.7–40.8)	44,849	14,388	32.08 (31.65–32.52)	39.64 (38.95–40.34)	25,335	8,944	35.3 (34.71–35.9)	41.37 (40.48–42.28)	75.73	**< 0.001**
Morelos	23,482	7,806	33.24 (32.64–33.85)	41.84 (40.84–42.87)	15,312	4,927	32.18 (31.44–32.92)	41.97 (40.67–43.31)	8,170	2,879	35.24 (34.2–36.29)	41.84 (40.23–43.5)	22.50	**< 0.001**
Nayarit	43,608	12,698	29.12 (28.69–29.55)	38.61 (37.9–39.33)	29,004	8,148	28.09 (27.58–28.61)	37.75 (36.87–38.64)	14,604	4,550	31.16 (30.41–31.91)	40.32 (39.09–41.59)	44.16	**< 0.001**
Nuevo Leon	240,809	80,127	33.27 (33.09–33.46)	40.9 (40.6–41.2)	152,486	49,968	32.77 (32.53–33.01)	40.9 (40.52–41.29)	88,323	30,159	34.15 (33.83–34.46)	40.93 (40.45–41.41)	47.79	**< 0.001**
Oaxaca	29,066	10,554	36.31 (35.76–36.87)	43.32 (42.44–44.21)	18,987	6,680	35.18 (34.5–35.87)	42.65 (41.55–43.78)	10,079	3,874	38.44 (37.49–39.39)	44.55 (43.1–46.05)	30.15	**< 0.001**
Puebla	137,465	65,729	47.82 (47.55–48.08)	52.6 (52.18–53.03)	85,639	39,651	46.3 (45.97–46.63)	51.94 (51.4–52.49)	51,826	26,078	50.32 (49.89–50.75)	53.83 (53.16–54.51)	208.92	**< 0.001**
Queretaro	94,432	41,517	43.96 (43.65–44.28)	49.13 (48.63–49.63)	57,965	24,670	42.56 (42.16–42.96)	48.34 (47.69–48.99)	36,467	16,847	46.2 (45.69–46.71)	50.42 (49.63–51.22)	120.24	**< 0.001**
Quintana Roo	37,371	12,673	33.91 (33.43–34.39)	40.64 (39.88–41.4)	23,747	7,803	32.86 (32.26–33.46)	40.15 (39.19–41.14)	13,624	4,870	35.75 (34.94–36.56)	41.67 (40.45–42.93)	32.19	**< 0.001**
San Luis Potosi	58,987	23,807	40.36 (39.96–40.76)	45.18 (44.59–45.78)	37,669	15,005	39.83 (39.34–40.33)	45.06 (44.31–45.81)	21,318	8,802	41.29 (40.63–41.95)	45.54 (44.57–46.53)	11.98	**< 0.001**
Sinaloa	196,686	48,641	24.73 (24.54–24.92)	32.46 (32.16–32.77)	128,424	30,644	23.86 (23.63–24.1)	31.56 (31.19–31.94)	68,262	17,997	26.36 (26.03–26.7)	34.14 (33.62–34.68)	150.01	**< 0.001**
Sonora	148,897	46,246	31.06 (30.82–31.29)	37.69 (37.33–38.05)	94,915	28,637	30.17 (29.88–30.46)	36.83 (36.38–37.28)	53,982	17,609	32.62 (32.22–33.02)	39.21 (38.61–39.81)	96.38	**< 0.001**
State of Mexico	537,766	263,076	48.92 (48.79–49.05)	53.12 (52.91–53.33)	332,806	159,116	47.81 (47.64–47.98)	52.59 (52.32–52.86)	204,960	103,960	50.72 (50.51–50.94)	54.03 (53.7–54.37)	430.34	**< 0.001**
Tabasco	43,470	11,822	27.2 (26.78–27.62)	36.03 (35.34–36.73)	28,608	7,430	25.97 (25.46–26.48)	35.93 (35.05–36.84)	14,862	4,392	29.55 (28.82–30.29)	36.56 (35.43–37.73)	63.32	**< 0.001**
Tamaulipas	95,927	26,630	27.76 (27.48–28.05)	38.5 (38.01–39)	63,804	17,227	27 (26.66–27.35)	38.41 (37.79–39.04)	32,123	9,403	29.27 (28.77–29.77)	38.75 (37.92–39.59)	55.00	**< 0.001**
Tlaxcala	9,854	4,667	47.36 (46.37–48.35)	51.89 (50.35–53.47)	6,188	2,854	46.12 (44.87–47.37)	51.58 (49.6–53.65)	3,666	1,813	49.45 (47.82–51.09)	52.58 (50.12–55.14)	10.26	**0.001**
Veracruz	162,180	51,659	31.85 (31.63–32.08)	39.51 (39.16–39.87)	104,091	31,833	30.58 (30.3–30.86)	38.75 (38.3–39.21)	58,089	19,826	34.13 (33.74–34.52)	40.95 (40.36–41.55)	216.27	**< 0.001**
Yucatan	47,502	15,154	31.9 (31.48–32.32)	40.28 (39.6–40.97)	30,278	9,338	30.84 (30.32–31.36)	40.5 (39.62–41.4)	17,224	5,816	33.77 (33.06–34.48)	40.15 (39.08–41.26)	43.26	**< 0.001**
Zacatecas	25,944	11,288	43.51 (42.9–44.11)	48.56 (47.64–49.5)	16,283	6,818	41.87 (41.11–42.63)	47.2 (46.04–48.39)	9,661	4,470	46.27 (45.27–47.27)	50.81 (49.29–52.37)	47.69	**< 0.001**

CI, confidence interval; SER, spherical equivalent refraction. The Chi-square test of independence was used to test the association between sex and the prevalence of myopia. A *p*-value < 0.05 was considered statistically significant.

[a]Age-adjusted prevalence to the World Health Organization (WHO) world standard population.

Supplementary information

Table S4. Crude and age-adjusted state-level prevalence estimates of low myopia among Mexican outpatients

State	Total individuals (*n*)	Low myopia (-6.0 D < SER ≤ -0.50 D)												
		General			Female				Male				χ^2	*p*-value
		n	Crude % (95% CI)	Age-adjusted[a] % (95% CI)	Total	*n*	Crude % (95% CI)	Age-adjusted[a] % (95% CI)	Total	*n*	Crude % (95% CI)	Age-adjusted[a] % (95% CI)		
Aguascalientes	53,003	20,035	37.8 (37.39–38.21)	41.64 (41.05–42.25)	33,475	12,318	36.8 (36.28–37.32)	41.02 (40.27–41.79)	19,528	7,717	39.52 (38.83–40.21)	42.76 (41.78–43.75)	38.81	**< 0.001**
Baja California	237,989	78,643	33.04 (32.86–33.23)	37.29 (37.02–37.56)	147,674	47,901	32.44 (32.2–32.68)	36.59 (36.25–36.94)	90,315	30,742	34.04 (33.73–34.35)	38.38 (37.94–38.83)	64.98	**< 0.001**
Baja California Sur	13,893	4,532	32.62 (31.84–33.41)	39.58 (38.37–40.83)	8,970	2,790	31.1 (30.15–32.07)	37.93 (36.43–39.49)	4,923	1,742	35.38 (34.05–36.74)	42.55 (40.48–44.72)	26.51	**< 0.001**
Campeche	11,260	3,876	34.42 (33.54–35.31)	42.45 (41.05–43.89)	6,870	2,351	34.22 (33.1–35.36)	42.81 (40.99–44.71)	4,390	1,525	34.74 (33.33–36.17)	42 (39.83–44.28)	0.32	0.573
Chiapas	73,934	22,723	30.73 (30.4–31.07)	37.87 (37.35–38.4)	46,352	13,828	29.83 (29.42–30.25)	37.53 (36.85–38.22)	27,582	8,895	32.25 (31.7–32.8)	38.54 (37.7–39.4)	47.44	**< 0.001**
Chihuahua	89,474	32,170	35.95 (35.64–36.27)	43.46 (42.96–43.96)	57,123	20,036	35.08 (34.68–35.47)	43.09 (42.46–43.72)	32,351	12,134	37.51 (36.98–38.04)	44.12 (43.3–44.95)	53.06	**< 0.001**
Coahuila	73,016	28,036	38.4 (38.04–38.75)	44.38 (43.83–44.92)	47,229	17,750	37.58 (37.15–38.02)	43.9 (43.23–44.59)	25,787	10,286	39.89 (39.29–40.49)	45.19 (44.29–46.11)	37.48	**< 0.001**
Colima	29,598	7,748	26.18 (25.68–26.68)	34.15 (33.34–34.97)	18,373	4,700	25.58 (24.95–26.22)	34.28 (33.23–35.35)	11,225	3,048	27.15 (26.33–27.99)	34.04 (32.78–35.34)	8.92	**0.003**
Durango	62,126	21,630	34.82 (34.44–35.19)	41.18 (40.61–41.76)	40,713	13,772	33.83 (33.37–34.29)	40.73 (40.02–41.46)	21,413	7,858	36.7 (36.05–37.35)	41.95 (40.99–42.92)	50.94	**< 0.001**
Guanajuato	168,612	67,070	39.78 (39.54–40.01)	45 (44.65–45.36)	104,987	40,002	38.1 (37.81–38.4)	43.99 (43.53–44.45)	63,625	27,068	42.54 (42.16–42.93)	46.75 (46.18–47.33)	326.19	**< 0.001**
Guerrero	35,753	8,482	23.72 (23.28–24.17)	33.31 (32.55–34.09)	23,562	5,405	22.94 (22.4–23.48)	32.94 (31.97–33.93)	12,191	3,077	25.24 (24.47–26.02)	34.14 (32.87–35.46)	23.50	**< 0.001**
Hidalgo	24,334	9,961	40.93 (40.32–41.56)	45.43 (44.52–46.37)	15,102	6,019	39.86 (39.07–40.64)	45.03 (43.84–46.24)	9,232	3,942	42.7 (41.69–43.72)	46.26 (44.79–47.78)	19.16	**< 0.001**
Jalisco	248,652	87,864	35.34 (35.15–35.52)	40.62 (40.34–40.91)	157,599	54,331	34.47 (34.24–34.71)	40.06 (39.7–40.42)	91,053	33,533	36.83 (36.51–37.14)	41.55 (41.1–42.02)	139.93	**< 0.001**
Mexico City	382,552	172,776	45.16 (45.01–45.32)	52.38 (52.12–52.64)	243,069	106,223	43.7 (43.5–43.9)	51.75 (51.41–52.08)	139,483	66,553	47.71 (47.45–47.98)	53.55 (53.12–53.97)	576.37	**< 0.001**
Michoacan	70,184	22,611	32.22 (31.87–32.56)	39.02 (38.49–39.57)	44,849	13,926	31.05 (30.62–31.48)	38.43 (37.74–39.12)	25,335	8,685	34.28 (33.7–34.87)	40.15 (39.28–41.05)	77.34	**< 0.001**
Morelos	23,482	7,639	32.53 (31.93–33.13)	40.99 (40–42.01)	15,312	4,826	31.52 (30.78–32.26)	41.17 (39.89–42.51)	8,170	2,813	34.43 (33.4–35.47)	40.93 (39.34–42.58)	20.60	**< 0.001**
Nayarit	43,608	12,477	28.61 (28.19–29.04)	37.97 (37.26–38.68)	29,004	8,013	27.63 (27.11–28.15)	37.15 (36.29–38.04)	14,604	4,464	30.57 (29.82–31.32)	39.56 (38.34–40.82)	41.10	**< 0.001**
Nuevo Leon	240,809	78,744	32.7 (32.51–32.89)	40.2 (39.91–40.5)	152,486	49,078	32.19 (31.95–32.42)	40.2 (39.82–40.58)	88,323	29,666	33.59 (33.28–33.9)	40.24 (39.77–40.72)	50.01	**< 0.001**
Oaxaca	29,066	10,219	35.16 (34.61–35.71)	42.03 (41.17–42.91)	18,987	6,455	34 (33.32–34.68)	41.3 (40.22–42.42)	10,079	3,764	37.34 (36.4–38.3)	43.35 (41.91–44.83)	32.37	**< 0.001**
Puebla	137,465	63,729	46.36 (46.1–46.62)	51.05 (50.64–51.47)	85,639	38,343	44.77 (44.44–45.11)	50.3 (49.76–50.84)	51,826	25,386	48.98 (48.55–49.41)	52.43 (51.77–53.11)	230.16	**< 0.001**
Queretaro	94,432	40,415	42.8 (42.48–43.11)	47.88 (47.38–48.37)	57,965	23,976	41.36 (40.96–41.77)	47.05 (46.41–47.7)	36,467	16,439	45.08 (44.57–45.59)	49.22 (48.44–50.01)	126.27	**< 0.001**
Quintana Roo	37,371	12,421	33.24 (32.76–33.72)	39.88 (39.14–40.65)	23,747	7,647	32.2 (31.61–32.8)	39.42 (38.46–40.4)	13,624	4,774	35.04 (34.24–35.85)	40.9 (39.69–42.15)	31.45	**< 0.001**
San Luis Potosi	58,987	23,160	39.26 (38.87–39.66)	44.02 (43.44–44.61)	37,669	14,578	38.7 (38.21–39.19)	43.86 (43.13–44.61)	21,318	8,582	40.26 (39.6–40.92)	44.44 (43.49–45.42)	13.84	**< 0.001**
Sinaloa	196,686	47,658	24.23 (24.04–24.42)	31.81 (31.51–32.11)	128,424	30,023	23.38 (23.15–23.61)	30.94 (30.56–31.31)	68,262	17,635	25.83 (25.51–26.16)	33.43 (32.9–33.96)	146.47	**< 0.001**
Sonora	148,897	45,175	30.34 (30.11–30.57)	36.84 (36.49–37.2)	94,915	27,937	29.43 (29.14–29.72)	35.97 (35.53–36.41)	53,982	17,238	31.93 (31.54–32.33)	38.39 (37.8–38.99)	101.69	**< 0.001**
State of Mexico	537,766	254,177	47.27 (47.13–47.4)	51.35 (51.14–51.56)	332,806	153,308	46.07 (45.9–46.23)	50.72 (50.46–50.99)	204,960	100,869	49.21 (49–49.43)	52.44 (52.11–52.77)	504.54	**< 0.001**
Tabasco	43,470	11,603	26.69 (26.28–27.11)	35.39 (34.71–36.09)	28,608	7,296	25.5 (25–26.01)	35.32 (34.45–36.22)	14,862	4,307	28.98 (28.25–29.72)	35.88 (34.76–37.03)	60.42	**< 0.001**
Tamaulipas	95,927	26,162	27.27 (26.99–27.56)	37.83 (37.34–38.33)	63,804	16,940	26.55 (26.21–26.89)	37.78 (37.16–38.4)	32,123	9,222	28.71 (28.21–29.21)	38.01 (37.19–38.84)	50.18	**< 0.001**
Tlaxcala	9,854	4,422	44.88 (43.89–45.86)	49.16 (47.66–50.7)	6,188	2,694	43.54 (42.3–44.78)	48.72 (46.79–50.74)	3,666	1,728	47.14 (45.51–48.77)	50.06 (47.66–52.56)	12.06	**< 0.001**
Veracruz	162,180	50,540	31.16 (30.94–31.39)	38.71 (38.36–39.07)	104,091	31,127	29.9 (29.63–30.18)	37.98 (37.53–38.43)	58,089	19,413	33.42 (33.04–33.8)	40.11 (39.52–40.7)	214.83	**< 0.001**
Yucatan	47,502	14,856	31.27 (30.86–31.69)	39.54 (38.87–40.23)	30,278	9,140	30.19 (29.67–30.71)	39.72 (38.84–40.62)	17,224	5,716	33.19 (32.48–33.9)	39.49 (38.42–40.58)	45.95	**< 0.001**
Zacatecas	25,944	10,915	42.07 (41.47–42.67)	47.05 (46.15–47.98)	16,283	6,563	40.31 (39.55–41.06)	45.57 (44.42–46.74)	9,661	4,352	45.05 (44.05–46.05)	49.51 (48.01–51.05)	55.93	**< 0.001**

CI, confidence interval; SER, spherical equivalent refraction.. The Chi-square test of independence was used to test the association between sex and the prevalence of low myopia. A *p*-value < 0.05 was considered statistically significant.

[a]Age-adjusted prevalence to the World Health Organization (WHO) world standard population.

Supplementary information

Table S5. Crude and age-adjusted state-level prevalence estimates of high myopia among Mexican outpatients

State	Total individuals (*n*)	High myopia (SER ≤ -6.0 D)												
		General			Female				Male					
		n	Crude % (95% CI)	Age-adjusted[a] % (95% CI)	Total	*n*	Crude % (95% CI)	Age-adjusted[a] % (95% CI)	Total	*n*	Crude % (95% CI)	Age-adjusted[a] % (95% CI)	χ^2	*p*-value
Aguascalientes	53,003	455	0.86 (0.78–0.94)	0.92 (0.84–1.02)	33,475	282	0.84 (0.75–0.95)	0.9 (0.79–1.02)	19,528	173	0.89 (0.76–1.03)	0.96 (0.82–1.12)	0.27	0.601
Baja California	237,989	1,664	0.7 (0.67–0.73)	0.78 (0.74–0.82)	147,674	1,008	0.68 (0.64–0.73)	0.74 (0.69–0.79)	90,315	656	0.73 (0.67–0.78)	0.83 (0.77–0.9)	1.55	0.214
Baja California Sur	13,893	88	0.64 (0.51–0.79)	0.73 (0.58–0.93)	8,646	59	0.68 (0.52–0.88)	0.78 (0.59–1.02)	4,673	29	0.62 (0.42–0.89)	0.79 (0.52–1.17)	0.24	0.626
Campeche	11,260	95	0.85 (0.69–1.04)	1.01 (0.81–1.26)	6,812	54	0.79 (0.6–1.03)	0.97 (0.72–1.32)	4,120	41	1 (0.72–1.35)	1.2 (0.85–1.66)	0.70	0.403
Chiapas	73,934	469	0.63 (0.58–0.69)	0.78 (0.71–0.86)	46,352	302	0.65 (0.58–0.73)	0.81 (0.71–0.92)	27,243	167	0.61 (0.52–0.71)	0.75 (0.63–0.88)	0.58	0.445
Chihuahua	89,474	602	0.68 (0.63–0.74)	0.82 (0.76–0.9)	56,483	376	0.67 (0.6–0.74)	0.82 (0.73–0.91)	31,972	226	0.71 (0.62–0.8)	0.83 (0.72–0.96)	0.50	0.478
Coahuila	73,016	539	0.74 (0.68–0.8)	0.82 (0.75–0.9)	47,229	360	0.76 (0.69–0.84)	0.84 (0.75–0.94)	25,787	179	0.69 (0.6–0.8)	0.78 (0.66–0.91)	1.06	0.304
Colima	29,598	171	0.58 (0.5–0.68)	0.73 (0.62–0.86)	18,157	110	0.61 (0.5–0.73)	0.75 (0.61–0.94)	11,075	61	0.55 (0.42–0.71)	0.69 (0.52–0.91)	0.37	0.543
Durango	62,126	481	0.77 (0.71–0.85)	0.87 (0.79–0.96)	40,713	316	0.78 (0.69–0.87)	0.87 (0.77–0.98)	21,084	165	0.78 (0.67–0.91)	0.9 (0.76–1.05)	0.01	0.940
Guanajuato	168,612	1,586	0.94 (0.9–0.99)	1.05 (1–1.11)	104,987	980	0.93 (0.88–0.99)	1.04 (0.97–1.11)	63,625	606	0.95 (0.88–1.03)	1.06 (0.98–1.15)	0.15	0.695
Guerrero	35,753	215	0.6 (0.52–0.69)	0.88 (0.76–1.03)	23,562	143	0.61 (0.51–0.71)	0.91 (0.74–1.12)	12,094	72	0.6 (0.47–0.75)	0.86 (0.66–1.11)	0.04	0.850
Hidalgo	24,334	250	1.04 (0.92–1.18)	1.1 (0.96–1.25)	14,905	157	1.05 (0.9–1.23)	1.1 (0.92–1.3)	9,114	93	1.02 (0.82–1.25)	1.1 (0.89–1.37)	0.06	0.809
Jalisco	248,652	1,921	0.77 (0.74–0.81)	0.85 (0.81–0.89)	157,599	1,222	0.78 (0.73–0.82)	0.85 (0.8–0.91)	91,053	699	0.77 (0.71–0.83)	0.85 (0.78–0.92)	0.04	0.833
Mexico City	382,552	6,018	1.57 (1.53–1.61)	1.74 (1.69–1.78)	243,069	3,906	1.61 (1.56–1.66)	1.79 (1.73–1.85)	139,483	2,112	1.51 (1.45–1.58)	1.66 (1.58–1.73)	4.93	**0.026**
Michoacan	70,184	721	1.03 (0.95–1.1)	1.22 (1.13–1.32)	44,849	462	1.03 (0.94–1.13)	1.21 (1.1–1.34)	25,335	259	1.02 (0.9–1.15)	1.22 (1.07–1.39)	0.01	0.921
Morelos	23,482	167	0.72 (0.62–0.84)	0.86 (0.72–1.02)	14,761	101	0.68 (0.56–0.83)	0.9 (0.72–1.11)	8,056	66	0.82 (0.63–1.04)	0.92 (0.7–1.21)	1.66	0.198
Nayarit	43,608	221	0.51 (0.44–0.58)	0.64 (0.56–0.75)	29,004	135	0.47 (0.39–0.55)	0.59 (0.49–0.72)	13,475	86	0.64 (0.51–0.79)	0.8 (0.63–1)	2.93	0.087
Nuevo Leon	240,809	1,383	0.57 (0.54–0.61)	0.7 (0.66–0.74)	152,486	890	0.58 (0.55–0.62)	0.7 (0.65–0.75)	88,323	493	0.56 (0.51–0.61)	0.68 (0.62–0.75)	0.64	0.425
Oaxaca	29,066	335	1.15 (1.03–1.28)	1.29 (1.15–1.45)	18,987	225	1.19 (1.04–1.35)	1.35 (1.16–1.56)	9,383	110	1.17 (0.96–1.41)	1.27 (1.03–1.56)	0.51	0.477
Puebla	137,465	2,000	1.45 (1.39–1.52)	1.55 (1.48–1.62)	85,639	1,308	1.53 (1.45–1.61)	1.64 (1.55–1.74)	51,826	692	1.34 (1.24–1.44)	1.4 (1.29–1.51)	8.31	**0.004**
Queretaro	94,432	1,102	1.17 (1.1–1.24)	1.25 (1.18–1.34)	57,965	694	1.2 (1.11–1.29)	1.29 (1.19–1.4)	36,467	408	1.12 (1.01–1.23)	1.2 (1.08–1.33)	1.19	0.274
Quintana Roo	37,371	252	0.68 (0.6–0.77)	0.76 (0.67–0.87)	22,809	156	0.68 (0.58–0.8)	0.78 (0.66–0.94)	13,523	96	0.71 (0.58–0.87)	0.78 (0.62–0.97)	0.29	0.588
San Luis Potosi	58,987	647	1.11 (1.03–1.2)	1.18 (1.09–1.28)	37,219	427	1.15 (1.04–1.26)	1.21 (1.09–1.34)	21,040	220	1.05 (0.91–1.19)	1.12 (0.97–1.28)	1.29	0.255
Sinaloa	196,686	983	0.5 (0.47–0.53)	0.66 (0.61–0.7)	128,424	621	0.48 (0.45–0.52)	0.63 (0.57–0.68)	67,120	362	0.54 (0.49–0.6)	0.73 (0.65–0.81)	1.96	0.162
Sonora	148,897	1,071	0.72 (0.68–0.76)	0.84 (0.79–0.9)	94,915	700	0.74 (0.68–0.79)	0.86 (0.79–0.93)	53,982	371	0.69 (0.62–0.76)	0.81 (0.73–0.91)	1.22	0.270
State of Mexico	537,766	8,899	1.65 (1.62–1.69)	1.77 (1.73–1.81)	332,806	5,808	1.75 (1.7–1.79)	1.87 (1.82–1.93)	204,960	3,091	1.51 (1.46–1.56)	1.6 (1.54–1.66)	43.80	**< 0.001**
Tabasco	43,470	219	0.5 (0.44–0.57)	0.64 (0.55–0.74)	28,360	134	0.47 (0.4–0.56)	0.62 (0.51–0.76)	14,247	85	0.6 (0.48–0.74)	0.71 (0.56–0.9)	2.09	0.148
Tamaulipas	95,927	468	0.49 (0.44–0.53)	0.67 (0.61–0.74)	63,804	287	0.45 (0.4–0.5)	0.63 (0.55–0.72)	31,766	181	0.57 (0.49–0.66)	0.75 (0.64–0.88)	5.68	**0.017**
Tlaxcala	9,854	245	2.49 (2.19–2.81)	2.73 (2.39–3.12)	6,188	160	2.59 (2.2–3.01)	2.86 (2.41–3.4)	3,666	85	2.32 (1.86–2.86)	2.52 (2.01–3.15)	0.68	0.411
Veracruz	162,180	1,119	0.69 (0.65–0.73)	0.8 (0.75–0.86)	104,091	706	0.68 (0.63–0.73)	0.78 (0.72–0.84)	58,089	413	0.71 (0.64–0.78)	0.84 (0.76–0.93)	0.58	0.445
Yucatan	47,502	298	0.63 (0.56–0.71)	0.75 (0.66–0.85)	29,939	198	0.66 (0.57–0.76)	0.79 (0.68–0.93)	17,028	100	0.59 (0.48–0.71)	0.68 (0.55–0.84)	0.95	0.330
Zacatecas	25,944	373	1.44 (1.3–1.59)	1.51 (1.36–1.68)	16,104	255	1.58 (1.4–1.79)	1.66 (1.45–1.89)	9,661	118	1.22 (1.01–1.46)	1.3 (1.07–1.57)	5.08	**0.024**

CI, confidence interval; SER, spherical equivalent refraction.. The Chi-square test of independence was used to test the association between sex and the prevalence of high myopia. A *p*-value < 0.05 was considered statistically significant.

[a]Age-adjusted prevalence to the World Health Organization (WHO) world standard population.

Supplementary information

Table S6. Univariable multinomial logistic regression analysis of variables associated with low and high myopia among Mexican outpatients

Variables	Myopia severity							
	Low myopia				High myopia			
	B	SE	RRR[a] (95% CI)	*p*-value	*B*	SE	RRR[a] (95% CI)	*p*-value
Age (years)	-0.044	0.0001	0.957 (0.957–0.957)	**< 0.001**	-0.0387	0.0003	0.962 (0.962–0.963)	**< 0.001**
Sex								
**Female**			**Reference**				**Reference**	
Male	0.1353	0.0023	1.145 (1.14–1.15)	**< 0.001**	0.0097	0.0112	1.01 (0.988–1.032)	0.3904
State of residence								
**State of Mexico**			**Reference**				**Reference**	
Aguascalientes	-0.4066	0.0094	0.666 (0.654–0.678)	**< 0.001**	-0.8393	0.0484	0.432 (0.393–0.475)	**< 0.001**
Baja California	-0.6181	0.0052	0.539 (0.534–0.544)	**< 0.001**	-1.1216	0.0269	0.326 (0.309–0.343)	**< 0.001**
Baja California Sur	-0.6383	0.0183	0.528 (0.51–0.548)	**< 0.001**	-1.2273	0.1076	0.293 (0.237–0.362)	**< 0.001**
Campeche	-0.5538	0.0201	0.575 (0.553–0.598)	**< 0.001**	-0.9119	0.1039	0.402 (0.328–0.493)	**< 0.001**
Chiapas	-0.7259	0.0084	0.484 (0.476–0.492)	**< 0.001**	-1.2552	0.0476	0.285 (0.26–0.313)	**< 0.001**
Chihuahua	-0.4892	0.0075	0.613 (0.604–0.622)	**< 0.001**	-1.1147	0.0423	0.328 (0.302–0.356)	**< 0.001**
Coahuila	-0.3831	0.0081	0.682 (0.671–0.693)	**< 0.001**	-0.983	0.0447	0.374 (0.343–0.408)	**< 0.001**
Colima	-0.9512	0.0135	0.386 (0.376–0.397)	**< 0.001**	-1.4133	0.0775	0.243 (0.209–0.283)	**< 0.001**
Durango	-0.5376	0.0089	0.584 (0.574–0.594)	**< 0.001**	-0.9905	0.0471	0.371 (0.339–0.407)	**< 0.001**
Guanajuato	-0.3214	0.0057	0.725 (0.717–0.733)	**< 0.001**	-0.7146	0.0275	0.489 (0.464–0.517)	**< 0.001**
Guerrero	-1.0823	0.0127	0.339 (0.33–0.347)	**< 0.001**	-1.407	0.0694	0.245 (0.214–0.281)	**< 0.001**
Hidalgo	-0.2715	0.0134	0.762 (0.743–0.782)	**< 0.001**	-0.5985	0.0645	0.55 (0.484–0.624)	**< 0.001**
Jalisco	-0.5147	0.005	0.598 (0.592–0.604)	**< 0.001**	-0.9853	0.0254	0.373 (0.355–0.392)	**< 0.001**
Mexico City	-0.0873	0.0043	0.916 (0.909–0.924)	**< 0.001**	-0.0925	0.0169	0.912 (0.882–0.942)	**< 0.001**
Michoacan	-0.6509	0.0086	0.522 (0.513–0.53)	**< 0.001**	-0.7447	0.039	0.475 (0.44–0.513)	**< 0.001**
Morelos	-0.6413	0.0142	0.527 (0.512–0.542)	**< 0.001**	-1.1104	0.0785	0.329 (0.282–0.384)	**< 0.001**
Nayarit	-0.8296	0.011	0.436 (0.427–0.446)	**< 0.001**	-1.5142	0.0685	0.22 (0.192–0.252)	**< 0.001**
Nuevo Leon	-0.6356	0.0051	0.53 (0.524–0.535)	**< 0.001**	-1.325	0.0291	0.266 (0.251–0.281)	**< 0.001**
Oaxaca	-0.5164	0.0126	0.597 (0.582–0.612)	**< 0.001**	-0.5808	0.0561	0.559 (0.501–0.624)	**< 0.001**
Puebla	-0.0408	0.0061	0.96 (0.949–0.972)	**< 0.001**	-0.1504	0.0251	0.86 (0.819–0.904)	**< 0.001**
Queretaro	-0.1919	0.0072	0.825 (0.814–0.837)	**< 0.001**	-0.4429	0.0323	0.642 (0.603–0.684)	**< 0.001**
Quintana Roo	-0.6098	0.0113	0.543 (0.532–0.556)	**< 0.001**	-1.1567	0.0643	0.315 (0.277–0.357)	**< 0.001**
San Luis Potosi	-0.3404	0.0089	0.711 (0.699–0.724)	**< 0.001**	-0.5643	0.0411	0.569 (0.525–0.616)	**< 0.001**
Sinaloa	-1.0558	0.0059	0.348 (0.344–0.352)	**< 0.001**	-1.5849	0.0338	0.205 (0.192–0.219)	**< 0.001**
Sonora	-0.7432	0.0063	0.476 (0.47–0.481)	**< 0.001**	-1.1333	0.0326	0.322 (0.302–0.343)	**< 0.001**
Tabasco	-0.9257	0.0112	0.396 (0.388–0.405)	**< 0.001**	-1.5449	0.0687	0.213 (0.186–0.244)	**< 0.001**
Tamaulipas	-0.8964	0.0078	0.408 (0.402–0.414)	**< 0.001**	-1.5671	0.0476	0.209 (0.19–0.229)	**< 0.001**
Tlaxcala	-0.0819	0.0207	0.921 (0.885–0.959)	**< 0.001**	0.3774	0.0663	1.458 (1.281–1.661)	**< 0.001**
Veracruz	-0.7049	0.006	0.494 (0.488–0.5)	**< 0.001**	-1.1627	0.0319	0.313 (0.294–0.333)	**< 0.001**
Yucatan	-0.7006	0.0103	0.496 (0.486–0.506)	**< 0.001**	-1.2539	0.0591	0.285 (0.254–0.32)	**< 0.001**
Zacatecas	-0.2172	0.0129	0.805 (0.785–0.825)	**< 0.001**	-0.2462	0.0536	0.782 (0.704–0.868)	**< 0.001**
Astigmatism severity								
**No astigmatism**			**Reference**				**Reference**	
Mild (-1.50 D < Cyl ≤ -0.75 D)	1.1888	0.0028	3.283 (3.265–3.302)	**< 0.001**	1.6671	0.0209	5.297 (5.084–5.518)	**< 0.001**
Moderate (-2.50 D ≤ Cyl ≤ -1.50 D)	2.2258	0.0039	9.261 (9.191–9.332)	**< 0.001**	3.6201	0.0186	37.34 (36.002–38.727)	**< 0.001**
Severe (Cyl < -2.50 D)	2.8917	0.0059	18.023 (17.815–18.234)	**< 0.001**	5.0823	0.0184	161.141 (155.445–167.047)	**< 0.001**
Eye care behavior								
**No prior eye examination/no eyeglassess use**			**Reference**				**Reference**	
Prior eye examination/no eyeglassess use	0.0887	0.0082	1.093 (1.075–1.11)	**< 0.001**	-0.2485	0.0625	0.78 (0.69–0.882)	**< 0.001**
No prior examination/eyeglassess use	0.2092	0.0054	1.233 (1.22–1.246)	**< 0.001**	1.027	0.0243	2.793 (2.663–2.929)	**< 0.001**
Prior examination/eyeglassess use	0.4499	0.0023	1.568 (1.561–1.575)	**< 0.001**	1.2556	0.0127	3.51 (3.424–3.598)	**< 0.001**
Diabetes								
**No**			**Reference**				**Reference**	
Yes	-0.8088	0.0045	0.445 (0.441–0.449)	**< 0.001**	-0.715	0.0231	0.489 (0.468–0.512)	**< 0.001**
High blood pressure								
**No**			**Reference**				**Reference**	
Yes	-1.0098	0.0042	0.364 (0.361–0.367)	**< 0.001**	-0.9002	0.0216	0.406 (0.39–0.424)	**< 0.001**

B, coefficients; CI, confident interval; D, diopters; SE, standard error; RRR, relative risk ratio.

[a]RRR estimated using univariable multinomial logistic regression. A *p*-value < 0.05 was considered statistically significant.

Supplementary information

Table S7. Multiple multinomial logistic regression analyses of variables associated with low and high myopia among Mexican outpatients

Variables	Myopia severity							
	Low myopia				High myopia			
	B	SE	RRR[a] (95% CI)	*p*-value	*B*	SE	RRR[a] (95% CI)	*p*-value
Age (years)	-0.0446	0.0001	0.956 (0.956–0.956)	**< 0.001**	-0.0354	0.0003	0.965 (0.965–0.966)	**< 0.001**
Sex								
**Female**			**Reference**				**Reference**	
Male	-0.0492	0.0028	0.952 (0.947–0.957)	**< 0.001**	-0.3403	0.0117	0.712 (0.695–0.728)	**< 0.001**
State of residence								
**State of Mexico**			**Reference**				**Reference**	
Aguascalientes	-0.3214	0.0111	0.725 (0.709–0.741)	**< 0.001**	-0.5827	0.0496	0.558 (0.507–0.615)	**< 0.001**
Baja California	-0.5981	0.0062	0.55 (0.543–0.557)	**< 0.001**	-0.9357	0.0276	0.392 (0.372–0.414)	**< 0.001**
Baja California Sur	-0.4257	0.0214	0.653 (0.626–0.681)	**< 0.001**	-0.6643	0.1094	0.515 (0.415–0.638)	**< 0.001**
Campeche	-0.4867	0.0235	0.615 (0.587–0.644)	**< 0.001**	-0.6574	0.1061	0.518 (0.421–0.638)	**< 0.001**
Chiapas	-0.3863	0.0098	0.68 (0.667–0.693)	**< 0.001**	-0.5706	0.0486	0.565 (0.514–0.622)	**< 0.001**
Chihuahua	-0.3316	0.0089	0.718 (0.705–0.73)	**< 0.001**	-0.7564	0.0432	0.469 (0.431–0.511)	**< 0.001**
Coahuila	-0.2499	0.0097	0.779 (0.764–0.794)	**< 0.001**	-0.6829	0.0456	0.505 (0.462–0.552)	**< 0.001**
Colima	-0.5911	0.0155	0.554 (0.537–0.571)	**< 0.001**	-0.6719	0.079	0.511 (0.438–0.596)	**< 0.001**
Durango	-0.31	0.0106	0.733 (0.718–0.749)	**< 0.001**	-0.5582	0.0483	0.572 (0.521–0.629)	**< 0.001**
Guanajuato	-0.1802	0.0068	0.835 (0.824–0.846)	**< 0.001**	-0.3946	0.0283	0.674 (0.638–0.712)	**< 0.001**
Guerrero	-0.8001	0.0147	0.449 (0.437–0.462)	**< 0.001**	-0.8675	0.0707	0.42 (0.366–0.482)	**< 0.001**
Hidalgo	-0.1007	0.0162	0.904 (0.876–0.933)	**< 0.001**	-0.2105	0.0666	0.81 (0.711–0.923)	**0.0016**
Jalisco	-0.3299	0.006	0.719 (0.711–0.727)	**< 0.001**	-0.551	0.026	0.576 (0.548–0.607)	**< 0.001**
Mexico City	0.0776	0.0052	1.081 (1.07–1.092)	**< 0.001**	0.1006	0.0176	1.106 (1.068–1.145)	**< 0.001**
Michoacan	-0.3552	0.0101	0.701 (0.687–0.715)	**< 0.001**	-0.2291	0.0403	0.795 (0.735–0.861)	**< 0.001**
Morelos	-0.3485	0.0167	0.706 (0.683–0.729)	**< 0.001**	-0.6433	0.0801	0.526 (0.449–0.615)	**< 0.001**
Nayarit	-0.5859	0.0128	0.557 (0.543–0.571)	**< 0.001**	-1.0467	0.0695	0.351 (0.306–0.402)	**< 0.001**
Nuevo Leon	-0.3934	0.0061	0.675 (0.667–0.683)	**< 0.001**	-0.7569	0.0297	0.469 (0.443–0.497)	**< 0.001**
Oaxaca	-0.2939	0.0151	0.745 (0.724–0.768)	**< 0.001**	-0.2232	0.0582	0.8 (0.714–0.897)	**< 0.001**
Puebla	0.0861	0.0074	1.09 (1.074–1.106)	**< 0.001**	0.0482	0.026	1.049 (0.997–1.104)	0.0636
Queretaro	-0.1167	0.0086	0.89 (0.875–0.905)	**< 0.001**	-0.3135	0.0332	0.731 (0.685–0.78)	**< 0.001**
Quintana Roo	-0.3839	0.0132	0.681 (0.664–0.699)	**< 0.001**	-0.6163	0.0654	0.54 (0.475–0.614)	**< 0.001**
San Luis Potosi	-0.1486	0.0108	0.862 (0.844–0.88)	**< 0.001**	-0.1584	0.0425	0.853 (0.785–0.928)	**< 0.001**
Sinaloa	-0.8236	0.0069	0.439 (0.433–0.445)	**< 0.001**	-1.0851	0.0344	0.338 (0.316–0.361)	**< 0.001**
Sonora	-0.6618	0.0075	0.516 (0.508–0.524)	**< 0.001**	-0.8757	0.0334	0.417 (0.39–0.445)	**< 0.001**
Tabasco	-0.5949	0.0129	0.552 (0.538–0.566)	**< 0.001**	-0.8397	0.0698	0.432 (0.377–0.495)	**< 0.001**
Tamaulipas	-0.5577	0.0091	0.573 (0.562–0.583)	**< 0.001**	-0.8768	0.0485	0.416 (0.378–0.458)	**< 0.001**
Tlaxcala	-0.0854	0.0251	0.918 (0.874–0.964)	**< 0.001**	0.4088	0.0699	1.505 (1.312–1.726)	**< 0.001**
Veracruz	-0.5205	0.0071	0.594 (0.586–0.603)	**< 0.001**	-0.7925	0.0327	0.453 (0.425–0.483)	**< 0.001**
Yucatan	-0.4483	0.0119	0.639 (0.624–0.654)	**< 0.001**	-0.7254	0.0604	0.484 (0.43–0.545)	**< 0.001**
Zacatecas	-0.1337	0.0156	0.875 (0.849–0.902)	**< 0.001**	-0.0659	0.0554	0.936 (0.84–1.044)	0.2344
Astigmatism severity								
**No astigmatism**			**Reference**				**Reference**	
Mild (-1.50 D < Cyl ≤ -0.75 D)	1.1963	0.0031	3.308 (3.288–3.328)	**< 0.001**	1.6198	0.021	5.052 (4.848–5.264)	**< 0.001**
Moderate (-2.50 D ≤ Cyl ≤ -1.50 D)	2.1864	0.0043	8.903 (8.828–8.978)	**< 0.001**	3.526	0.0189	33.989 (32.756–35.268)	**< 0.001**
Severe (Cyl < -2.50 D)	2.4707	0.0064	11.831 (11.684–11.979)	**< 0.001**	4.6097	0.0189	100.453 (96.794–104.251)	**< 0.001**
Eye care behavior								
**No prior eye examination/no eyeglassess use**			**Reference**				**Reference**	
Prior eye examination/no eyeglassess use	0.0323	0.0096	1.033 (1.014–1.052)	**< 0.001**	-0.2996	0.0634	0.741 (0.655–0.839)	**< 0.001**
No prior examination/eyeglassess use	0.477	0.0066	1.611 (1.591–1.632)	**< 0.001**	1.1072	0.0252	3.026 (2.88–3.179)	**< 0.001**
Prior examination/eyeglassess use	0.6069	0.0029	1.835 (1.824–1.845)	**< 0.001**	1.1754	0.0132	3.239 (3.157–3.324)	**< 0.001**
Diabetes								
**No**			**Reference**				**Reference**	
Yes	-0.0155	0.0054	0.985 (0.974–0.995)	**0.0043**	-0.0844	0.0256	0.919 (0.874–0.966)	**0.001**
High blood pressure								
**No**			**Reference**				**Reference**	
Yes	-0.2313	0.0051	0.793 (0.786–0.801)	**< 0.001**	-0.3449	0.0244	0.708 (0.675–0.743)	**< 0.001**

B, coefficients; CI, confident interval; D, diopters; SE, standard error; RRR, relative risk ratio.

[a]RRR estimated using multivariable multinomial logistic regression. A *p*-value < 0.05 was considered statistically significant.
